# Biogeography of community canopy leaf traits and their links to global forest photosynthesis

**DOI:** 10.1126/sciadv.adk2998

**Published:** 2026-07-10

**Authors:** Feng Jiang, Bernhard Schmid, Xucai Pu, Chengjun Ji, Zehao Shen, Zhiyao Tang, Xiangping Wang, Chengyang Zheng, Biao Zhu, Peter B. Reich, Jingyun Fang, Zhiheng Wang

**Affiliations:** ^1^ Institute of Ecology, State Key Laboratory of Vegetation Structure, Function and Construction (VegLab), College of Urban and Environmental Sciences, Peking University, Beijing, China.; ^2^ Remote Sensing Laboratories, Department of Geography, University of Zürich, Zürich, Switzerland.; ^3^ School of Ecology and Nature Conservation, Beijing Forestry University, Beijing, China.; ^4^ Institute for Global Change Biology and School for Environment and Sustainability, University of Michigan, Ann Arbor, MI 48109, USA.; ^5^ Department of Forest Resources, University of Minnesota, St Paul, MN 55108, USA.

## Abstract

Leaf traits influence biotic interactions and ecosystem functions in forests. However, biogeographic drivers of leaf traits remain highly uncertain, limiting their integration into global vegetation models. Using a global dataset of forest plots, we show that community canopy leaf traits align along three dimensions: leaf economy, leaf density, and nitrogen-to-phosphorus (N:P) ratio. Changes in these trait dimensions across forest communities were driven by different variables: leaf economy was primarily shaped by the proportion of deciduous trees, leaf density by soil available P and temperature, and leaf N:P ratio by temperature. These three leaf trait dimensions together explained 60.2% of the variation in ecosystem-scale forest maximum photosynthesis, with the leaf N:P ratio showing the strongest association, followed by the dimensions of leaf economy and leaf density. Forests with moderate leaf N:P ratios and more acquisitive traits had higher photosynthesis than other forests. Our findings highlight the potential of community canopy leaf traits in predicting biogeographic variation in ecosystem-scale forest functioning.

## Teaser

Multidimensional leaf traits shape forest photosynthesis.

## INTRODUCTION

Forests play a pivotal role in the global carbon cycle, covering 31% of the global land area (*1*) and contributing disproportionately to terrestrial gross primary production (*2*) and total plant biomass (*3*). Tree leaves in forests serve as the primary interface for photosynthesis and respiration (*4*, *5*), and mediate interactions with biotic agents such as herbivores and pathogens (*6*). Leaf traits, such as specific leaf area (SLA) and nitrogen content per dry mass (Nmass), are closely linked to plant growth strategies (*7*–*9*), plant–herbivore interactions (*6*, *10*), and nutrient stoichiometry (*11*–*13*) and serve as critical inputs for global vegetation models (*14*, *15*). Therefore, accurately mapping leaf traits in forest communities and identifying their drivers are essential for predicting forest functions such as photosynthesis and enhancing global vegetation models (*14*, *15*).

Latitudinal gradients of leaf traits have been a key topic in studying the strength of biotic interactions (*16*–*18*), nutrient limitation (*12*, *13*), and functional biogeography (*7*, *12*). For example, the critical leaf trait Nmass is suggested to be more conservative (e.g., lower Nmass) at lower latitudes due to lower nutrient availability (*12*) and for the protection against higher herbivore pressure (*17*). However, empirical studies have often reported weak and sometimes inconsistent latitudinal patterns across different leaf traits (*12*, *19*–*22*). To explain geographic patterns of leaf traits, several climate- and soil-related hypotheses have been developed (*12*). For example, previous studies suggest that more acquisitive traits (e.g., higher Nmass and SLA) are favored in colder environments, where plants invest more in N-rich enzymes to maintain metabolism (*12*), while more conservative traits (e.g., lower Nmass and SLA) are common in tropical regions with old, weathered, and nutrient-poor soils (*12*). Although global-scale analyses have shown that climate and soil properties can influence geographic patterns of leaf traits (*19*–*21*, *23*–*26*), their explanatory power—particularly for leaf economic traits—is relatively low. These weak and inconsistent associations between leaf traits and latitude, climate, or soil properties reduce the accuracy of trait distribution models solely relying on environmental predictors (*24*, *27*–*31*). Consequently, despite many efforts to map global leaf traits, large discrepancies remain among existing maps (*32*), limiting their uses in global vegetation models and in predicting forest responses to climate change.

Besides the potential influences of environmental variables, leaf traits also vary between co-occurring tree phenological types. Evergreen trees, which retain leaves year-round, typically exhibit more conservative traits (*9*). In contrast, deciduous trees, which shed their leaves seasonally, generally have more acquisitive traits (*33*, *34*). Therefore, despite considerable trait variation within each group (*34*), community leaf traits differ strongly with the relative abundance of deciduous versus evergreen trees (hereafter, community deciduousness) according to the so-called “evergreen-deciduous hypothesis” (*12*). Given that community deciduousness is generally associated with large-scale environmental gradients (e.g., seasonality) (*35*–*37*), trait mapping models based on environments may have captured the effect of community deciduousness on leaf traits to some extent. However, beyond environments, community deciduousness may also be shaped by biotic feedbacks, where recruits matching the dominant leaf phenology in a community tend to have higher survival and growth rates (*38*). For example, such feedbacks have led to the emergence of alternative stable states in high-latitude forests, where forests are usually dominated by either deciduous or evergreen trees (*38*). Therefore, including community deciduousness may improve trait mapping models (*24*, *27*–*31*). Moreover, the influences of community deciduousness on leaf trait variation may be stronger at higher latitudes, where trait differences between deciduous and evergreen trees are more pronounced (*39*). However, previous global-scale analyses have rarely integrated community deciduousness in mapping leaf traits (*4*, *12*, *20*, *25*, *39*, *40*).

Leaf traits are generally considered to be closely linked to leaf photosynthetic rates (*4*, *5*, *41*). The leaf economics spectrum theory posits that acquisitive leaves—characterized by high SLA, Nmass and phosphorus content per dry mass (Pmass), and low leaf tissue density (LD)—are associated with higher photosynthetic capacity and more rapid carbon return (*4*). In part, the is because both leaf Nmass and Pmass reflect key components of photosynthesis such as enzymatic capacity (e.g., Rubisco) and energy metabolism (*12*, *42*). Therefore, the balance between nitrogen and phosphorus in leaves (e.g., leaf N:P ratio) may also be closely related to photosynthetic performance (*42*). Overall, numerous studies at both local and global scales have demonstrated relationships between these commonly measured leaf traits and leaf-level photosynthetic rates (*4*, *41*). However, it remains unclear whether these relationships observed at the leaf level can be consistently observed at the community level, particularly in forest ecosystems (*43*). This uncertainty limits our ability to determine whether spatially-explicit maps of leaf traits can reliably inform vegetation physiological processes and ecosystem primary productivity in global vegetation models (*44*).

**Fig. 1.**
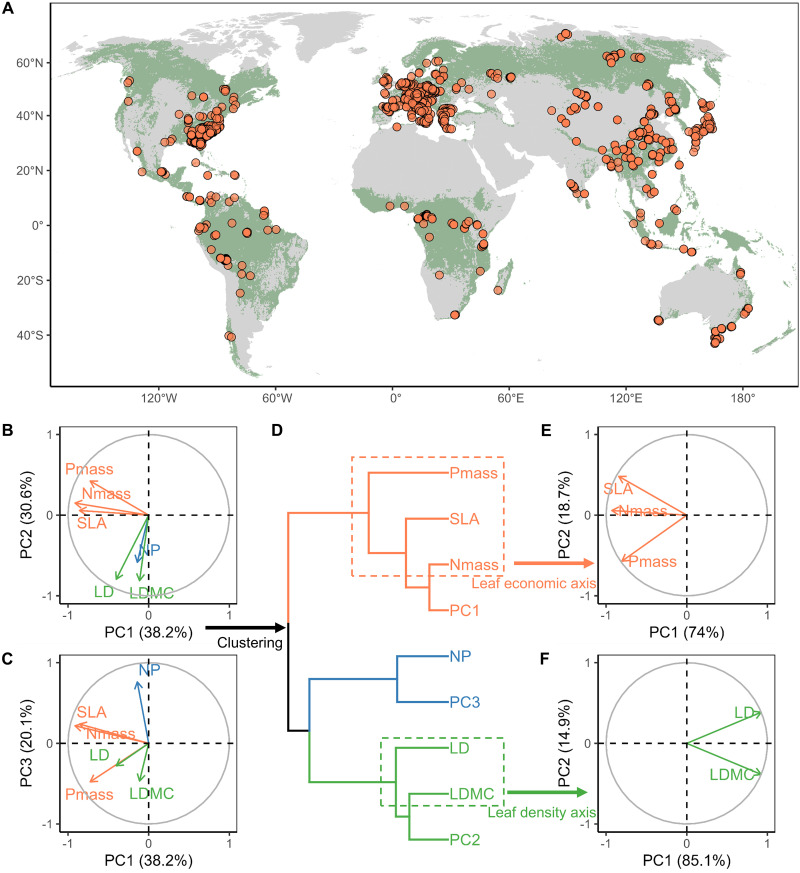
Dimensions of community canopy leaf traits in forests. (**A**) distribution of forest plots used in this study (*N* = 2797); dark green areas are forest regions defined as land with more than 10% tree cover; (**B** and **C**) principal component analysis for community canopy leaf traits; (**D**) clustering of these traits together with the extracted principal components (PCs) in (B) and (C); (**E** and **F**) principal component analyses for leaf economic and leaf density traits separately, where the first axis is used to characterize the axes of leaf economy and leaf density, respectively. All trait variables are log-transformed and standardized (z-scores) before principal component analysis. SLA, specific leaf area, mm^2^/mg; Nmass, leaf nitrogen per dry mass, mg/g; Pmass, leaf phosphorus per dry mass, mg/g; LDMC, leaf dry matter content, g/g; LD, leaf tissue density, g/cm^3^; NP, leaf N:P ratio, g/g.

In this study, we used 2797 forest plots around the globe and integrated environmental variables and community deciduousness to map community canopy leaf traits (i.e., species abundance-weighted mean trait values) of forests and to identify the drivers underlying their geographic patterns (Fig. 1A). The community canopy leaf traits included SLA, Nmass, Pmass, leaf dry matter content (LDMC), LD (leaf dry mass divided by leaf volume), and leaf N:P ratio. These leaf traits were selected because they have been widely measured and represent key functional traits frequently studied in leaf economics theory (*4*), plant–herbivory interactions (*45*), and ecological stoichiometry (*12*, *46*). First, we assessed the correlations between these six community canopy leaf traits. Second, we evaluated whether community deciduousness could explain additional variation in leaf traits beyond climate and soil, and used it together with environmental layers to produce global maps of the six leaf traits. Third, we quantified the dominant drivers of the geographic patterns of these leaf traits. Finally, we evaluated whether and which traits could predict global forest maximum photosynthesis.

## RESULTS

### Dimensions of community canopy leaf trait variation

Principal component analysis (PCA) using the six leaf traits (SLA, Nmass, Pmass, LDMC, LD, and N:P ratio) showed that the first three principal components (PCs) explained 88.9% of the variation in community leaf traits across global forest communities (Fig. 1, B to D). PC1 (explaining 38.2% variation, here we defined the leaf economic traits) was most strongly associated with SLA, Nmass, and Pmass and reflected the ‘fast-slow’ economic spectrum, ranging from communities with more acquisitive strategies (fast growth) to those with more conservative strategies (slow growth) (table S1). PC2 (explaining 30.6% variation, here we defined the leaf density traits) was strongly associated with LDMC and LD (table S1). This suggests that leaf density traits are relatively independent of leaf economic traits at the community level in forests. Finally, PC3 (explained 20.1% of variation) was strongly associated with leaf N:P ratios (table S1). To better isolate the effects of economic traits and density traits from stoichiometry, we conducted PCAs for leaf economic traits and density traits separately (Fig. 1, E and F). In our subsequent main analyses, we demonstrate these three dimensions of leaf trait variations using the PC1 axis of leaf economic traits alone (74% variation in SLA, Nmass, and Pmass), the PC1 axis for leaf density traits alone (85.1% variation in LDMC and LD), and the leaf N:P ratio as single variable, respectively.

### Global mapping of community canopy leaf traits

**Fig. 2.**
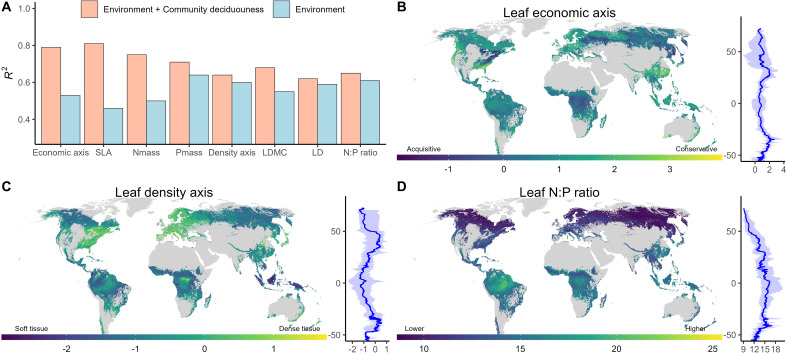
Global distributions of community canopy leaf traits in forests. (**A**) goodness of fit based on 10-fold cross-validation for two models: environment and community deciduousness as predictors; and only environmental predictors. (**B** to **D**) maps of leaf economic axis (the PC1 of SLA, Nmass, and Pmass, Fig. 1E), leaf density axis (the PC1 of LDMC and LD, Fig. 1F), and leaf N:P ratio. Plots on the right of panels (B) to (D) show the latitudinal gradients of leaf traits (median with 5% and 95% quantiles).

To map the global distributions of leaf traits across global forest communities, we used random forest models to fit the relationship between each community canopy leaf trait (or each trait dimension) and a set of predictors, including 27 climatic variables, 13 soil variables, 2 topographic variables, and community deciduousness (i.e., the proportion of deciduous tree abundance; see Materials and Methods). Including community deciduousness derived from the field survey data consistently improved model performance compared to models based on environmental variables alone. Among all leaf traits, including deciduousness led to the largest increase in model performance for SLA (Δ*R*^2^ = 0.35) and the smallest for the leaf N:P ratio (Δ*R*^2^ = 0.03) (Fig. 2A). Our models performed well, with *R*^2^ values ranging from 0.62 for LD to 0.81 for SLA (Fig. 2A and fig. S1). Relative to previous analyses based on either community- or species-level data (*19*, *20*), our high model performance is mainly attributable to the inclusion of community deciduousness, which showed the strongest relationships with leaf economic traits among the predictors examined. In addition, the use of non-parametric machine-learning models and the simultaneous inclusion of multiple predictors can also increase the model performance. This high model performance is particularly advantageous for trait mapping.

Global trait maps showed that the leaf economic axis, the leaf density axis, and each single trait representing these two trait dimensions except Pmass did not exhibit strong monotonic trends from low to high latitudes (Fig. 2, B and C, and figs. S2 and S3). In contrast, a clear declining trend was observed for the canopy leaf N:P ratio and an increasing trend for Pmass (Fig. 2D and fig. S2C). Along the leaf economic axis (Fig. 2B), the most acquisitive forests are located between 42° and 48°N, particularly in northeastern America, eastern Europe, and northeastern Asia. In contrast, the most conservative forests are found at latitudes of 30°–35° in both hemispheres. This pattern largely reflects the relatively conservative strategies of evergreen broad-leaved forests occurring in the transition zone between tropical and temperate forests. For example, subtropical laurel forests and Mediterranean forests often experience relatively harsh environmental conditions (e.g., seasonal drought or climatic variability), and their evergreen leaves are typically thick, leathery, or sclerophyllous (*47*). High-latitude forests in the northern hemisphere (30°–48°) vary greatly in community canopy leaf economy, ranging from highly acquisitive forests in more mesic eastern America to highly conservative forests in western Europe and western America (Fig. 2B). Along the leaf density axis, the densest leaves are found in temperate forests of northeastern America, most regions of western Europe, and of East Asia, whereas relatively less dense leaves occur in the tropical rainforests of Southeast Asia (Fig. 2C). Not surprisingly, LDMC and LD showed similar geographic patterns as the leaf density axis (fig. S2, D and E).

### Global drivers of community canopy leaf traits

To evaluate the global drivers of forest community leaf traits, we applied three methods, each contributing a complementary perspective (see Materials and Methods). First, we used random forest models to assess the relative importance of three predictor groups (climate, soil, and community deciduousness) in shaping community canopy leaf traits. Second, we conducted sensitivity analyses based on the random forest models to examine how the dominant drivers varied among geographic regions and to identify which variables were most important at different latitudes. Third, to determine the relative importance of individual variables, we used random forest models and selected 11 environmental variables with low collinearity that were expected to be tightly associated with leaf traits according to previous studies (*4*, *12*, *19*, *25*, *29*).

**Fig. 3.**
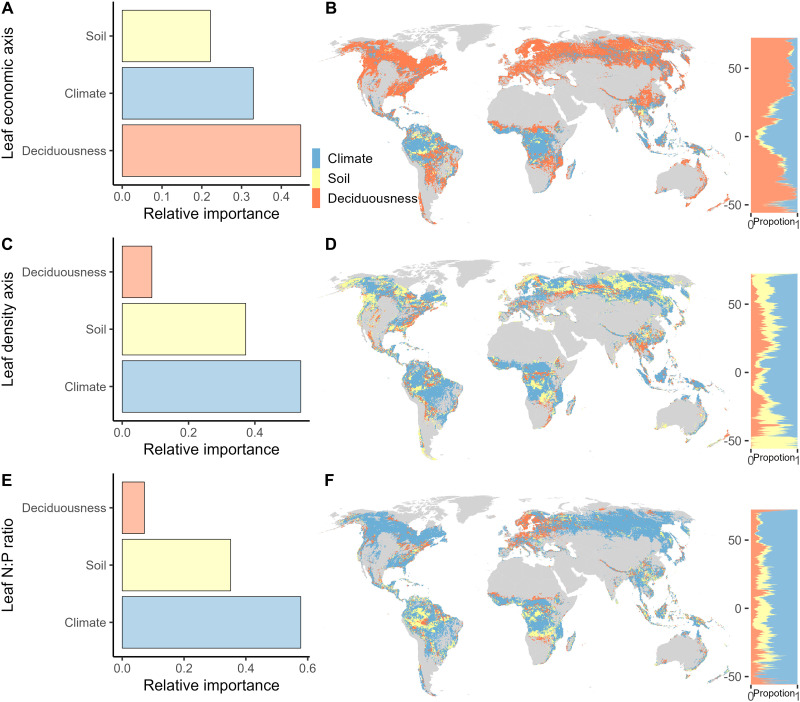
Relative importance of predictor groups shaping community canopy leaf traits in forests. (**A, C,** and **E**) relative importance of three predictor groups in random forest models (*N* = 2797). (**B, D,** and **F**) maps of the dominant drivers. Figures on the right of panels (B), (D), and (F) show the proportion of each dominant driver along with the latitudinal gradient (i.e., the number of grid cell for one dominant driver divided by the total number of grid cells in each 0.1-degree latitudinal bin).

**Fig. 4.**
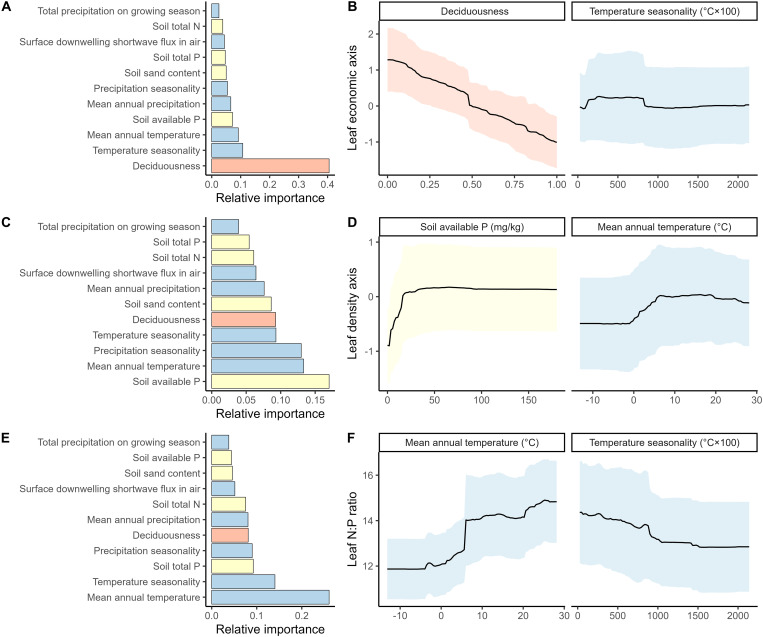
Relative importance of individual predictors shaping community canopy leaf traits in forests. (**A, C,** and **E**) relative importance of individual predictors based on random forest models (*N* = 2797). (**B, D,** and **F**) partial dependence plots (±standard deviation) showing the relationships between the top two predictors and leaf traits; these plots show the marginal effects of each predictor on the response variable, while holding other variables constant. The names and units of the x-axis variables are shown at the top of each panel.

We found that community deciduousness was the most important variable shaping the geographic variation in the leaf economic axis, followed by climate and soil properties (Figs. 3A and 4A). In general, forests with higher proportions of deciduous trees had more acquisitive leaf traits (Fig. 4B). The relative importance of community deciduousness and environmental variables varied across different forest biomes. Specifically, deciduousness emerged as the dominant driver in extratropical forests (>25°N and >25°S), whereas climate was the main drivers in low-latitude forests (Fig. 3B). These patterns were also observed for the determinants of the geographic variation in two individual leaf economic traits, i.e., SLA and Nmass (figs. S4, A and B, and S5, A and B). In contrast, another leaf economic trait, Pmass, was jointly influenced by climate and deciduousness; higher leaf Pmass was observed in forests with greater proportion of deciduous trees and lower temperature (figs. S4C and S5C).

Climate and soil properties jointly influenced the geographic variation in the community canopy leaf density axis, with soil available P and mean annual temperature as the two most important variables (Figs. 3, C and D, and 4C). Forests at higher soil available P and temperature tended to have denser leaf tissue (Fig. 4D). The main drivers of the geographic variation in the two individual traits representing the leaf density dimension, i.e., LDMC and LD, differed from that of the overall density axis. Specifically, soil available P and deciduousness were the two most important variables explaining the global distribution of LDMC; communities with high soil available P and a low proportion of deciduous trees had higher LDMC (fig. S5D). In addition to the effects of soil available P and community deciduousness, climate also played an important role in shaping LDMC variation at low latitudes (fig. S4D). In contrast, LD variation was more strongly driven by temperature and climate seasonality, particularly in boreal forests (fig. S4E). Overall, global communities in warmer regions had higher LD values (fig. S5E).

The leaf N:P ratio was primarily influenced by climate, especially at high latitudes (Fig. 3, E and F). Among all climate variables, temperature and its seasonality were the strongest predictors, with warmer and seasonally more stable-temperature forests having higher leaf N:P ratios (Fig. 4, E and F).

### Community deciduousness mediates community canopy trait–environment relationships

**Fig. 5.**
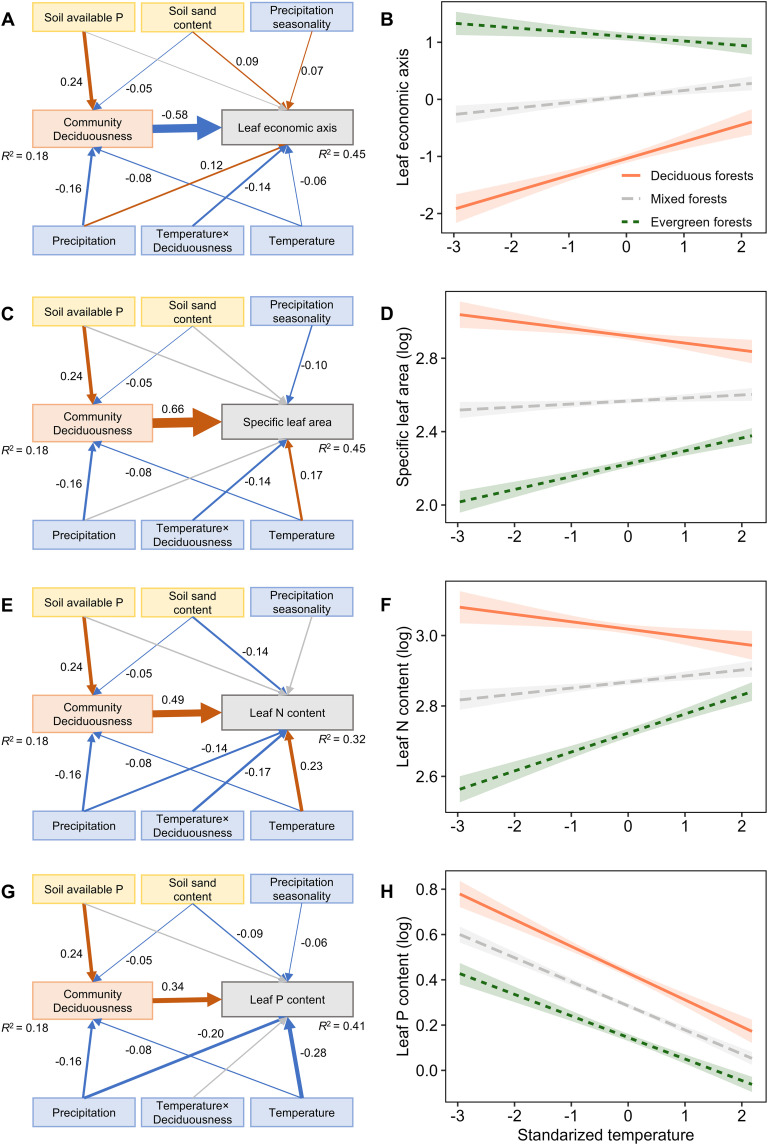
Community deciduousness mediates trait–environment relationships for community canopy leaf economic traits (both composite and individual traits) in forests. (**A, C, E,** and **G**) results of structural equation modelling (shared model fit across three models: *Fisher’s C* = 0.117, *P* = 0.943; *N* = 2797). Path coefficients are standardized: brown lines indicate significant positive relationships (*P* < 0.05), blue lines significant negative relationships, and gray lines non-significant relationships; line thickness reflects the strength of the relationship. (**B, D, F,** and **H**) interaction plots show that the relationships between temperature and leaf traits vary across different levels of community deciduousness (0.04, 0.51, and 0.99) modeled by simple linear models.

To disentangle the mechanisms underlying trait–environment relationships, we conducted structural equation models (SEM) to determine whether environmental variables influence trait variation directly due to environmental filtering or indirectly via their effects on community deciduousness. Given that community deciduousness had strong effects only on the geographic variation in leaf economic traits (SLA, Nmass, Pmass, and their composite PC1 axis), we restricted the SEM analysis to these traits. Among the five selected climatic and soil variables which have shown the strong relationships with leaf traits (Fig. 4 and fig. S6), soil available P and temperature seasonality showed the strongest influence on community deciduousness (standardized path coefficient = 0.24 and 0.30), followed by mean annual precipitation (−0.16) and temperature (−0.08) (Fig. 5).

Soil available P is associated with leaf economic traits through indirect rather than direct pathways. Forests with higher soil available P tend to have higher community deciduousness, which in turn relates to more acquisitive leaf traits (e.g., higher SLA) (Fig. 5). In contrast, precipitation seasonality is associated with leaf economic traits mainly through a relatively weak direct pathway, with higher seasonality associated with more conservative strategies. In addition to these two environmental variables, soil sand content, precipitation, and temperature are related to leaf economic traits through both direct and indirect pathways. For soil sand content, its association with deciduousness is relatively weak (−0.05), reflected in a weak indirect pathway to be associated with community leaf traits via community deciduousness. Consequently, soil sand content is associated with leaf economic traits (particularly for Nmass and Pmass) primarily through direct pathways, with higher sand content associated with lower Nmass and Pmass.

Although SEMs are based on these correlative associations between variables, they suggest the following causal hypotheses. Temperature and precipitation are major drivers of leaf economic traits (Fig. 5). Higher precipitation promotes more conservative community canopy leaf traits (e.g., lower Nmass and Pmass), both directly and indirectly by reducing the proportion of deciduous species in the community. In contrast to precipitation, the direct and indirect effects of temperature on leaf economic traits are often opposite. For example, increasing temperature directly increases SLA and Nmass, but also promotes a higher proportion of evergreen species, which tends to reduce SLA and Nmass. As a result, the overall effects of temperature on these traits are weakened. For Pmass, however, the direct and indirect effects of temperature act in the same direction, strengthening the overall negative effect of temperature (Fig. 5G). Temperature and community deciduousness also interactively influence leaf economic traits, particularly SLA and Nmass. In communities dominated by deciduous species, SLA and Nmass decrease with increasing temperature, whereas in evergreen-dominated communities these traits increase with temperature (Fig. 5, D and F). The results of SEMs conducted with temperature seasonality instead of mean annual temperature were similar to those obtained using mean annual temperature (fig. S7).

### Relationships between community canopy leaf traits and maximum forest photosynthesis

**Fig. 6.**
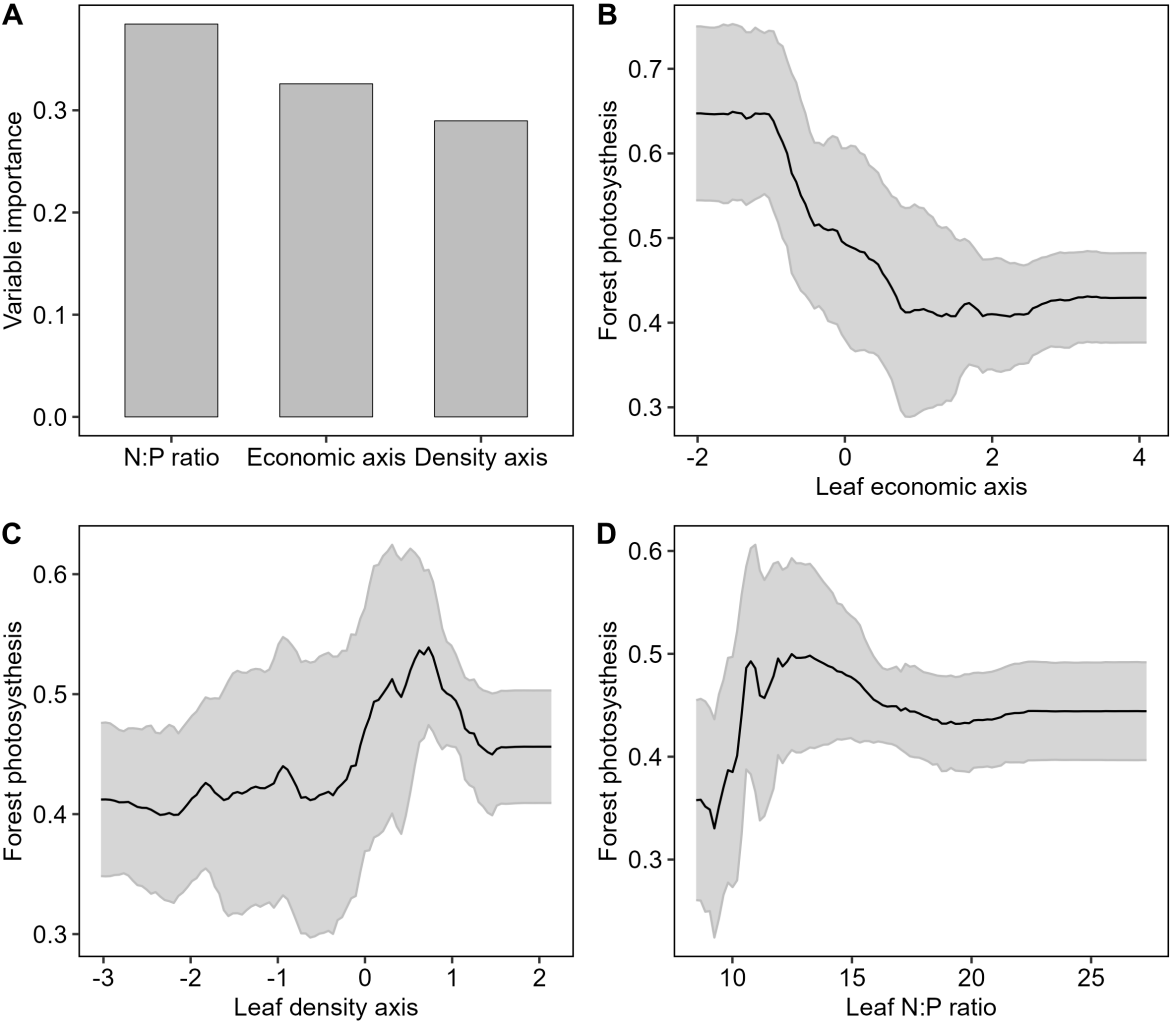
Relationships between community canopy leaf traits and maximum forest photosynthesis. **A,** variable importance of three community canopy trait dimensions in explaining forest maximum photosynthesis, represented by the monthly maximum of sun-induced fluorescence (SIF). Together, these three traits explained 60.2% of the variance in SIF values in the random forest model (*N* = 666,931 pixels). **B-D,** partial dependence plots (±standard deviation) for leaf economic axis, leaf density axis and leaf N:P ratio.

Forest photosynthetic capacity is a key driver of gross primary productivity, and its geographic pattern may be strongly related to the geographic patterns of leaf traits (*48*) (fig. S8; see Materials and Methods). To assess the relationships between community leaf traits and maximum forest photosynthesis, we fitted a multivariate random forest model using three trait dimensions (leaf economic axis, leaf density axis, and N:P ratio) as predictors and the monthly maximum of sun-induced fluorescence (SIF, the highest SIF in any given month) as the response variable and a proxy for forest maximum photosynthesis (*49*). While the three leaf trait dimensions together explained 60.2% of the variation in SIF values, the leaf N:P ratio showed the strongest association with SIF, followed by the leaf economic and leaf density axes (Fig. 6A). Forest photosynthesis was higher in forests with a moderate leaf N:P ratio (about 12), more acquisitive leaves, and moderate tissue density than in other forests (Fig. 6, B to D). These patterns remained unchanged when using multiple regression (slopes = −0.08, 0.10, and 0.03 for the leaf economic axis, leaf density axis, and N:P ratio, respectively). Univariate analyses showed similar results (fig. S9), with leaf N:P ratio and SLA being the two most important variables, explaining 22.2% and 16.0% of the variation, respectively.

## DISCUSSION

### Distinct drivers shape multidimensional community canopy leaf traits

We found that community deciduousness explained additional geographic variation in community canopy leaf traits beyond environmental variables, especially for leaf economic traits such as SLA and Nmass. These results provide support for the widely assumed but rarely tested “evergreen–deciduous hypothesis” (*12*, *19*), which posits that the relative abundance of deciduous versus evergreen trees influences the global distribution of community canopy leaf traits. The effect of community deciduousness is more pronounced in high-latitude forests, where shorter growing seasons favor distinct adaptation strategies (*9*). Deciduous trees typically show more acquisitive traits and shorter leaf lifespan in response to shorter growing seasons at higher latitudes (*50*). In contrast, evergreen trees tend to invest in longer-lived leaves to minimize carbon loss and maintenance costs under these same conditions (*50*). Even across different populations within evergreen species, shorter growing seasons tend to drive trees to increase leaf longevity but decrease Nmass (*51*). Consequently, the trait divergence between deciduous and evergreen trees increases with latitude (*39*, *50*, *52*), amplifying the influence of community deciduousness on community canopy leaf economic traits at high latitudes. High-latitude forests (when not evergreen-deciduous mixtures) thus exhibit either the most acquisitive leaves in deciduous broadleaf forests or the most conservative leaves in evergreen needleleaf forests. In contrast, in low-latitude forests, such as subtropical forests, leaf phenology is not as strongly correlated with leaf lifespan as in high latitudes because leaf lifespan can vary widely within the groups of evergreen and deciduous species, with some evergreen species having shorter leaf lifespans than some deciduous species (*5*, *33*). Overall, the mixture of evergreen and deciduous trees in temperate forests may weaken the latitudinal patterns of the leaf economic traits at the community level. Our findings also suggest that global vegetation models should consider this latitudinal variation in trait differences between evergreen and deciduous trees.

Although each individual leaf economic trait varies mainly along the integrated leaf economic axis, there remains other variation among individual traits. Especially, while Pmass was positively correlated with SLA and Nmass, their predicted maps diverged. Compared with SLA and Nmass, Pmass exhibited a clear monotonic change along the latitudinal gradient, which supports previous studies (*12*, *23*). These results suggest that different economic traits are still shaped by different drivers at different scales. For example, Pmass was more strongly influenced by climate than by community deciduousness. Even within climatic variables, temperature also showed contrasting effects on Pmass relative to their effects on SLA and Nmass. In agreement with previous findings (*25*, *26*, *46*), Pmass tends to be lower in warm and humid forests where soil P content is low (Fig. 5G), which is in line with the “soil substrate age hypothesis” proposed in previous literature (*12*). According to this hypothesis, tropical soils are generally older and more strongly weathered, leading to increased phosphorus leaching and, consequently, stronger P limitation compared with temperate regions.

Our results indicated that leaf density traits are weakly correlated with leaf economic traits at the community level, which likely reflects the fundamental differences in their adaption to environments. In contrast to the economic traits SLA and Nmass, the leaf density traits LDMC and LD are primarily shaped by environmental conditions and only weakly related to community deciduousness. Specifically, soil variables play an important role in shaping the geographic patterns of leaf density traits (*53*, *54*). For example, higher soil available P can substantially increase LDMC, consistent with findings from recent regional studies (*53*). Abundant available soil P can alleviate nutrient limitation, allowing plants to allocate more fixed carbon to structural components such as cell walls and cuticles, which may further contribute to increased LDMC and LD (*53*).

A strong latitudinal gradient was observed for the leaf N:P ratio, which is in line with previous findings (*12*, *23*, *29*). If leaf N:P ratio is used only as a proxy to indicate relative trends in nutrient limitation (*12*), our results suggest that high-latitude forests tend to be more N-limited, whereas tropical forests—particularly those in the Amazon—tend to be more P-limited. This geographic pattern of relative strength in soil nutrient limitations aligns with results from nutrient resorption efficiency studies (*55*) and supports the “soil substrate age hypothesis” (*12*). Our findings also suggest that the latitudinal gradient in leaf N:P ratio is mainly driven by the decrease in leaf P content with decreasing latitudes, while the N content remains relatively constant. Overall, our findings suggest that climate-driven variation in soil nutrients shapes the biogeographic patterns of plant nutrient limitation.

The leaf N:P ratio showed the strongest association with maximum forest photosynthesis, with photosynthesis peaking at intermediate N:P values (approximately 12). This pattern suggests that both relatively low levels of leaf N and P may constrain photosynthesis, as photosynthetic processes require both nitrogen-rich enzymes and phosphorus-rich energy compounds (*12*). In addition, intermediate N:P ratios may indicate relatively weaker soil N and P limitation (*12*). However, this interpretation should be treated with caution, as the thresholds of N:P used to infer nutrient limitation can vary across regions and among species (*56*). Furthermore, the monotonic relationships observed between forest photosynthesis and leaf economics spectrum traits are consistent with our expectations and align with the widely documented relationships between leaf-level traits and photosynthetic rates (*4*, *39*), suggesting that these relationships are robust across scales. Among individual leaf economic traits, we found that SLA, rather than Nmass and Pmass, was the strongest predictor of the maximum forest community photosynthesis. Given the strong association between SLA and community deciduousness, the prevalence of temperate deciduous forests with high SLA and moderate leaf N:P ratio likely enhances annual vegetation productivity, particularly in regions such as eastern North America and western Europe (*2*). We found that forest communities with intermediate tissue strength exhibited the highest photosynthetic capacity, suggesting that leaves may achieve a balance between resistance to herbivory and investment in photosynthetic function (*10*). These results further indicate that leaf density traits are largely independent of leaf economic traits at the community level and contribute independently to vegetation photosynthesis. Our findings suggest that integrating multidimensional leaf traits into environment-based models can enhance predictions of forest ecosystem functioning.

There are some caveats to our study. First, although we compiled a global forest plot database from multiple sources, tropical forests are underrepresented compared with temperate forests. In addition, many plots required trait imputation, particularly for leaf density traits, which may increase the uncertainty of trait predictions particularly in tropical regions. Increasing the number of directly measured traits and incorporating more tropical plots would further improve the robustness of our results. Second, we did not consider plastic variation within species along environmental gradients. However, incorporation of within species variation would likely amplify trait-geography, trait-environment and trait-trait relationships (*51*, *57*), because the available evidence suggests that within-species patterns generally mirror cross-species patterns (*51*, *57*, *58*). Third, we evaluated community-weighted mean trait values for all co-occurring trees, but the geographic patterns and drivers of leaf traits may differ among species groups with different leaf habits (e.g., evergreen vs. deciduous, and needleleaf vs. broadleaf). Therefore, distinguishing these differences will be important for future studies. Finally, our study focused on a limited set of leaf traits due to our emphasis on commonly considered leaf economic traits and the availability of trait observations. While other traits may also play important roles in linking leaf traits to vegetation distribution and photosynthesis, assessing their contributions would require additional trait measurements in future studies.

### Implications

Integrating community deciduousness with climatic and soil variables provides a promising framework for monitoring both historical and contemporary changes in the distributions of community-scale canopy leaf economic traits in global forests. Despite considerable variation in traits among species within and across geographic gradients within evergreen and deciduous groups, the evergreen-deciduous framework remains a valuable and persistent tool because its simple bifurcation of phenological types is highly informative and far easier to apply than measuring leaf longevity. Conversely, our leaf trait maps can also be used to predict and map community deciduousness. These maps may further improve our understanding of global biome patterns (*31*). Our findings suggest that accurately projecting future changes in community canopy leaf traits will be more feasible via first forecasting shifts in the relative composition of evergreen versus deciduous tree species within communities (*35*). However, studies relying on environmental variables to model community deciduousness so far had limited predictive power—for example, with *R*^2^ values of only 0.59 and 0.29 for broadleaved and needleleaf deciduous forests, respectively (*35*). Incorporating biological mechanisms such as plant–soil feedback may provide more accurate predictions of community deciduousness (*38*).

Considering the critical role of leaf traits in mediating plant–herbivore interactions, our findings challenge the latitudinal gradient hypothesis of biotic interactions (*18*), which posits that plants in lower latitudes should have more conservative and denser leaves to resist higher herbivory pressure. In contrast to this expectation, we did not find clear latitudinal patterns in the identified leaf trait dimensions leaf economy and leaf density, nor in the individual leaf economic traits SLA and Nmass. This divergence between our findings and the latitudinal gradient hypothesis of biotic interactions may be due to the overlooked role of deciduousness in shaping geographic variations in leaf economic traits. Given that deciduous and evergreen species may exhibit contrasting latitudinal gradients in leaf economic traits (*39*, *50*), we propose that they should be evaluated separately when examining plant–herbivore interactions (*59*). Recent studies suggest that some commonly measured leaf traits (like those used in our study) may not fully reflect global-scale herbivore defense strategies (*60*), and future research should consider functional traits related to secondary metabolism for better representation of plant responses to biotic pressures. However, our findings suggest that even for secondary metabolite traits, differences between deciduous and evergreen species should be explicitly considered (*59*).

By compiling a global forest plot database together with plant functional trait data, environmental variables, and community deciduousness, we mapped community canopy leaf traits and identified their drivers and consequences. Our results reveal that community canopy leaf traits vary along three relatively independent dimensions: the leaf economic spectrum, leaf tissue density, and leaf N:P ratio. These three dimensions of the multivariate trait space are shaped by distinct dominant drivers: leaf economic traits by community deciduousness, leaf tissue density by local climatic and soil variables, and leaf N:P ratios by climate (in particular temperature and water availability). The strong relationships between leaf economic traits and community deciduousness help answer two long-standing questions in functional biogeography: (*1*) the lack of latitudinal gradients of leaf economic traits, and (*2*) the limited ability of environmental variables to predict global distributions of leaf economic traits. We suggest that community deciduousness—an increasingly accessible trait via remote sensing—should be integrated into global trait distribution models. This will enhance our ability to evaluate historical and future changes in forest trait composition, and thus help better predict forest ecosystem functions such as photosynthesis, gross and net primary productivity under future climate change scenarios.

## MATERIALS AND METHODS

### Vegetation data

Our forest inventory plot data were compiled from three sources: our own forest plot data in China (*61*), published literature (*62*, *63*), and the sPlotOpen database (*64*). The China dataset includes 1,300 forest plots, each measuring 600 m^2^, and covers most forest types across mainland China. For both the China dataset and the literature-based plots, we included only plots where all trees with a diameter at breast height (DBH) ≥ 10 cm were measured. Finally, we excluded plots in which more than 30% of tree abundance could not be assigned to known species. Since these two sources provided limited coverage in North America and Europe, we supplemented them with forest plots from those regions in sPlotOpen. To ensure consistency, we filtered the sPlotOpen data to include only natural forests surveyed after 1970, with plot size larger than 100 m^2^. Since sPlotOpen generally reports species relative abundance without DBH thresholds and may include both woody and herbaceous species in forest plots, we retained only tree species and recalculated their relative abundance within each plot. To determine the tree species in the sPlotOpen dataset, we matched each species with the global tree species list (*65*). This step allowed us to focus on canopy tree species that dominate forest biomass. These criteria resulted in 6,038 plots. To reduce potential oversampling in these regions, we randomly selected 1,000 forest plots from these full data in sPlotOpen. Although this threshold is somewhat arbitrary, it is of a similar order of magnitude to our other plot dataset (1,797 plots), thereby ensuring reasonable comparability. Finally, our analysis included 2797 forest plots (table S2 and Fig. 1A).

### Community canopy leaf traits

In this study, we analyzed six leaf traits: specific leaf area (SLA, mm^2^/mg, leaf area per dry mass), leaf nitrogen content per dry mass (Nmass, mg/g), leaf phosphorus content per dry mass (Pmass, mg/g), leaf dry matter content (LDMC, g/g, leaf dry mass divided by leaf fresh mass), leaf tissue density (LD, g/cm^3^, leaf dry mass divided by leaf volume), and leaf nitrogen-to-phosphorus ratio (N:P ratio, g/g). Especially, the leaf N:P ratio was included because this trait can carry additional ecophysiological information beyond Nmass and Pmass (*12*), and we indeed found that the N:P ratio showed relationships independent of both Nmass and Pmass (Fig. 1). These traits are fundamental for understanding leaf economic strategies (*4*), stoichiometry (*12*), and interactions between leaves and herbivores or pathogens (*6*). In addition, they are commonly measured and key leaf economic traits with extensive coverage (*66*). Some other key traits, such as leaf longevity, *in situ* maximum net photosynthetic capacity, leaf maximum carboxylation capacity and intercellular CO_2_ concentration (*4*, *7*, *41*, *67*, *68*), may also be important due to their functional link with leaf economics. However, such trait data are rarely available, and a large proportion of species and plots would require trait gap-filling, particularly in tropical regions. Leaf size was also not included, mainly because it is often decoupled from leaf economic traits and its relationship with photosynthetic rate remains unclear (*19*), although it may still influence vegetation productivity (*44*). Overall, our study aimed to assess whether the widely used leaf economic traits and N:P ratio can effectively predict forest photosynthesis and to elucidate their relationships, rather than to try to find which leaf traits can best predict forest photosynthesis. Trait data were obtained from the TRY database (version 6) (*66*), and observations with an error risk score greater than three were excluded to ensure data quality. Species-level trait means were calculated for all plant species.

To maximize trait coverage across species, we impute missing values using the *mutate* function in the *funcspace* package (*69*). To provide more trait information for the process of imputing, we also included other 9 leaf traits (leaf area, leaf thickness, leaf texture, leaf carbon nitrogen ratio, leaf water content, leaf potassium content, leaf nitrogen isotope signature, photosynthesis rate per leaf dry mass, and leaf chlorophyll content per leaf dry mass). These traits are considered to be associated with leaf economic spectrum traits and leaf N:P ratio (*4*, *70*). In this study, we used these traits only as auxiliary variables to impute our focal leaf traits, and their inclusion improves the accuracy of trait gap-filling compared to using only the focal traits (*70*). We did not analyze these auxiliary variables because they are less commonly measured than our focal traits and therefore typically have smaller sample sizes (*66*), leading to potentially greater uncertainty compared with the core traits examined in this study. To assess the influence of trait imputation on our results, we reanalyzed the data using only plots with non-imputed trait values. The results were consistent with those obtained using the imputed trait dataset (table S3 and figs. S10 to S14). In particular, the maps of traits predicted from both datasets are highly consistent (SLA: *R*^2^ = 0.88; Nmass: 0.80; Pmass: 0.92; LDMC: 0.58; LD: 0.44; N:P: 0.81; *N* = 667,938 pixels).

For each forest plot, we calculated the community-weighted mean of each trait based on species abundance, using the following formula

$$\text{Community}-\text{weighted trait}=\sum_{i=1}^{n}{\text{trait}}_{i}*p_{i}$$ (1)

Where ${\text{trait}}_{i}$ and $p_{i}$ are the trait value and the relative abundance for species *i*. For sPlotOpen plots, the species relative abundance was estimated as the relative cover of each species. For most of the remaining plots (92%), relative abundance was calculated using the relative basal area of each species. In plots where basal area data were unavailable, we used importance values; if these were also unavailable, relative abundance was calculated based on stem density (number of individuals). These different methods for estimating species relative abundance are unlikely to substantially influence our results, as species relative basal area is strongly correlated with both relative importance values and relative stem density (*r* = 0.94 and 0.87, respectively; *N* = 4541 and 6899 species–plot combinations in our dataset where both metrics are available). Plots with more than 40% of total tree abundance without trait values were excluded from further analysis. Although different taxonomic groups (e.g., gymnosperms and angiosperms) may exhibit distinct trait–environment relationships, our objective was to assess patterns and drivers of community canopy trait values. Moreover, a substantial proportion of trait variation was explained by our predictors, suggesting that not explicitly accounting for these group differences is unlikely to affect the robustness of our results.

### Community deciduousness

We compiled leaf habit information (deciduous, evergreen, and semi-evergreen) of each tree species from TRY version 6 (*66*), Flora of China and The World Flora Online (https://www.worldfloraonline.org/). For species lacking leaf habit information, we extracted genus-level leaf habit data from the World Flora Online and assigned it to the corresponding species in our plot data. We calculated community deciduousness as the proportion of deciduous trees within each plot.

### Environmental variables

To map leaf traits and identity their drivers, we included 19 bioclimatic variables along with growing degree days (heat sum above 5°C), growing season length, growing season temperature, vapor pressure deficit, surface downwelling shortwave radiation, and growing season precipitation, all obtained from the CHELSA database (*71*). We also included aridity index and potential evapotranspiration from reference (*72*), elevation (original records, when available) and slope from reference (*73*), soil available phosphorus (0–20 cm) from reference (*74*), and soil total phosphorus (0–30 cm) from reference (*75*). In addition, we used 11 other soil physical and chemical properties (0–30 cm) from the SoilGrids 250 m dataset (*76*).

### Community canopy leaf trait correlations

To evaluate the correlations among community canopy leaf traits, we used principal component analysis (PCA). All community canopy leaf traits were log-transformed to improve normality as much as possible and then standardized. To evaluate the similarity among variables (all leaf traits and the first three PCs), we performed hierarchical clustering based on the absolute Pearson correlation coefficients. A dissimilarity matrix was generated by computing one minus the absolute value of each correlation coefficient, so that stronger correlations yielded smaller dissimilarity values. The clustering was performed using the average linkage method. The results revealed that traits varied along three primary dimensions: leaf economic traits, density traits, and N:P ratio. To better represent these dimensions in subsequent analyses, we performed separate PCAs for leaf economic traits (SLA, Nmass, and Pmass) and for leaf density traits (LDMC and LD), respectively.

### Geospatial mapping of leaf traits

To map the global distribution of leaf traits, we used random forest models to model the leaf economic axis (i.e., the PC1 of leaf economic traits, Fig. 1F), the leaf density axis (i.e., the PC1 of leaf density traits, Fig. 1E), leaf N:P ratio, and other raw community canopy leaf traits. Random forest models are well suited for prediction with large numbers of predictors as they are generally robust to multicollinearity and can effectively capture complex interactions among predictors (*77*). These advantages enable random forest models to achieve strong predictive performance when sample sizes are sufficient, and make them widely used in previous geo-mapping studies (*35*, *78*). All variables including climate, soil, topography, and field plot-based community deciduousness were included as predictors (table S4). Field plot-based deciduousness was used here because community deciduousness typically varies substantially at local scales, although it is correlated with satellite-derived values (*79*) (*r* = 0.38). To determine the optimal hyperparameters, we performed a random grid search with 10-fold cross-validation. The number of trees varied from 20 to 500 (20, 50, 100, 250, 500) and the minimum leaf size from 1 to 20 (1, 2, 5, 10, 20). The sample rate was 0.632, where each tree was trained on a bootstrap sample comprising approximately 63.2% of the original data. We also defined the candidate number of variables considered at each split as the floor of total predictor number multiplied by 0.05, 0.15, 0.25, 0.333, and 0.4. The best hyperparameter combinations were selected based on the highest coefficient of determination. Finally, we used the best-performing model to generate spatial predictions of leaf traits and assessed model performance using the 10-fold cross-validated *R*^2^. All models were implemented using the *h2o* package in R-4.3.1 (*80*, *81*).

To estimate the uncertainty in our global leaf trait predictions, we applied a bootstrap procedure, randomly sampling 2797 forest plots with replacement and stratified by biome (*82*). Biome classifications for each plot were matched using reference (*83*). This resampling process was repeated 100 times, generating 100 bootstrap datasets to train separate random forest models. These models were then used to produce 100 global prediction maps for each leaf trait. From these maps, we calculated the standard deviation of trait values for each grid cell to reflect the uncertainty of predicted trait values. Our predictions focused exclusively on forests which was defined as regions with more than 10% tree cover following the Food and Agriculture Organization of the United Nations; we used the tree cover data in 2010 accessed from reference (*79*). The proportion of deciduous trees in each forested grid cell was also obtained from reference (*79*), defined as the sum of the proportions of deciduous needleleaf and broadleaf trees (fig. S16A). The dataset of satellite-derived deciduous tree proportions was produced by integrating the ESA CCI land cover time series with high-resolution auxiliary datasets, including Landsat-derived tree cover and canopy height, resulting in spatially explicit annual maps of 14 plant functional types (*79*). To generate high-resolution trait maps, we used global layers of environmental variables and satellite-based deciduous tree proportions at a 0.1° (~11 km) resolution. For predictor layers with coarser (e.g., soil available phosphorus) or finer (e.g., land cover) resolutions, we resampled or aggregated them to match the 0.1° resolution.

To evaluate the impact of incorporating community deciduousness from field survey data on model performance, we fitted two random forest models. The first model included only environmental variables as predictors, while the second model included both environmental variables and field survey-based community deciduousness (Fig. 2A). We then compared the model performance (*R*^2^) between the two models.

To evaluate the influence of spatial autocorrelation on model performance (*84*), we first examined the spatial autocorrelation of model residuals using semivariograms and found that noticeable spatial autocorrelation occurred only at relatively short distances (within several tens of kilometers; fig. S15). To account for this, we performed spatial block cross-validation using the hclust function to cluster plots with buffer distances of 10 km, 50 km, and 100 km. We then evaluated the effects on model performance (*R*^2^) and found only minor reductions (e.g., for a 50 km buffer: leaf economic axis *R*^2^ = 0.63, leaf density axis *R*^2^ = 0.41, leaf N:P ratio *R*^2^ = 0.40; table S5).

### Relative importance of predictors in shaping community canopy leaf traits

To evaluate the relative importance of different predictor groups—climate, soil, and community deciduousness—in explaining the geographic patterns of leaf traits in global forests, we used random forest models. Compared to variance partitioning analysis based on linear models, random forest models can more readily accommodate a large number of predictors, capture nonlinear relationships, and estimate their relative importance (*35*). In addition, they can capture complex interactions among predictors, such that variable importance may implicitly reflect both main effects and interaction effects (*77*). We did not include topographic variables in these analyses because they represent more indirect environmental influences. To minimize the impact of multicollinearity on variable importance estimates, we conducted PCAs separately for climatic and soil variables and selected the first six PCs for climate and soil (explaining 90.7% and 85.4% of the variance, respectively). Variance inflation factors for all three predictor groups were ≤3.54, indicating sufficient independence. The relative importance of each predictor was obtained using the *h2o.varimp* function and is based on the reduction in node impurity (Gini index) contributed by each variable. The corresponding percentage represents each variable importance relative to the total importance of all predictors.

To evaluate the geographic patterns of dominant drivers—climate, soil, and community deciduousness—on leaf trait predictions, we conducted a sensitivity analysis (*85*). First, we built a full model using all predictor variables (the full model). Second, we removed one group of predictors (e.g., climate) and trained a new model (the sub-model). Third, we generated two sets of predicted leaf trait maps: one from the full model and one from the sub-model. Fourth, for each grid cell, we quantified the influence of the removed predictor group by calculating the relative deviation in predicted trait values

$${\text{Deviation}}_{\text{climate}}=\frac{|{\text{prediction}}_{\left[\text{full model}\right]}-{\text{prediction}}_{\left[\mathrm{sub}-\text{model}\right]}|}{|{\text{prediction}}_{\left[\text{full model}\right]}|}$$ (2)

Finally, we repeated this procedure for each predictor group. For each grid cell, we compared the relative deviations across all groups and identified the group with the highest deviation as the dominant driver. All random forest models in this section were implemented using the h2o package, and hyperparameter selection followed the same procedures described in the section on leaf trait mapping. As random forest models do not assume data normality, we used untransformed trait values.

To further identify the strongest individual predictors of leaf traits, we evaluated their relative importance using random forest models. To reduce multicollinearity, we selected key variables for leaf traits that showed low redundancy based on hierarchical clustering using the ClustOfVar package (*86*). We finally selected 11 variables with relatively low variance inflation factor (VIFs ranging from 1.3 to 2.0, except for mean annual temperature and temperature seasonality, which had VIFs of 5.66 and 5.19, respectively): community deciduousness, mean annual temperature, mean annual precipitation, temperature seasonality, precipitation seasonality, total precipitation on growing season, surface downwelling shortwave flux in air, soil available P, soil total P, soil total N and soil sand content.

### Direct and indirect associations of climate and soil to community canopy leaf traits

To distinguish whether environments influenced leaf traits by environmental filtering (e.g., physiological constraints) directly or indirectly via changing community deciduousness, we used structural equation modeling implemented with the *piecewiseSEM* package in R (*87*). Within the structural equation models, we used simple linear regressions to model the relationships between leaf traits and predictors (environments and community deciduousness) (fig. S6), and used generalized linear models (GLMs) with a binomial distribution to model the relationship between community deciduousness and environmental variables. For environmental variables, we selected three climatic variables (mean annual temperature, precipitation, and precipitation seasonality) and two soil variables (soil available P and sand content) that were key predictors for leaf traits (Fig. 4 and fig. S5) (*25*, *40*). Because the relationships between mean annual temperature and leaf traits may differ between evergreen and deciduous trees, we also included an interaction term between temperature and community deciduousness in the simple linear model. Because mean annual temperature and temperature seasonality were strongly correlated (*r* = −0.83; fig. S6), and temperature seasonality may strongly influence community deciduousness, we repeated the structural equation modeling using temperature seasonality in place of mean annual temperature. Because our focus was on how temperature influences leaf traits indirectly via community deciduousness (Fig. 4), we moved the results including temperature seasonality to the supplementary materials (fig. S7). Because community deciduousness showed strong influences on only leaf economic traits (SLA, Nmass, and Pmass; Fig. 4 and fig. S5), we focus analyses on these three traits. To improve normality of the functional trait distributions, we log-transformed SLA, Nmass, and Pmass.

### Predicting vegetation photosynthetic activity using leaf trait maps

One of the key goals of mapping plant functional traits is to improve their integration into global vegetation models and predict ecosystem functions. To evaluate whether community canopy trait maps can predict forest photosynthesis, we fitted a multivariate random forest model using three trait dimensions (leaf economic axis, leaf density axis, and N:P ratio) as predictors and the monthly maximum of sun-induced fluorescence (SIF, the highest SIF in any given month) as the response variable and a proxy for forest maximum photosynthesis in a location. SIF is a novel remote sensing–based vegetation index that reflects photosynthetic activity and is closely linked to gross primary productivity (GPP) (*88*).

We used a recently published SIF dataset with a spatial resolution of 0.05°, which provides monthly SIF values from 1995 to 2023 (*49*). For our analysis, we selected data from the years 1996, 2000, and 2005 to match the time frame of our most forest plot surveys. To assess how forest leaf functional traits (i.e., those generated in our study) influence SIF, we extracted the maximum monthly SIF values (i.e., the highest SIF in any given month in a given year) for each of the selected years—capturing peak photosynthetic activity during the growing season in high-latitude forests—and averaged them to produce a global SIF map. Annual GPP has been shown to be largely controlled by the length of the growing season and the photosynthetic capacity of vegetation, with leaf traits likely serving as key drivers of the latter (*48*). Using SIF data, GPP data (2000 and 2005) (*89*), and random forest models, we found that leaf traits were more strongly associated with peak photosynthetic activity and GPP (i.e., maximum SIF and monthly GPP) than with mean SIF or annual GPP after controlling for growing season length (fig. S8). Therefore, in this study we focus on evaluating the relationships between leaf traits and peak forest photosynthetic capacity. Finally, we aggregated this map to a 0.1° resolution to match the spatial resolution of our trait maps. In addition to the multivariate analysis, we also conducted univariate random forest models for each trait map and SIF value to evaluate the variation in SIF explained by individual traits.

All figures were generated using the R statistical software.
