# Supplementary material for: Biogeography of community canopy leaf traits and their links to global forest photosynthesis
Source: Sci Adv. 2026 Jul 10;12(28):eadk2998. doi: 10.1126/sciadv.adk2998 (PMC13353372; doi:10.1126/sciadv.adk2998)
Supplement: Supplementary file 1 — Figs. S1 to S16 Tables S1 to S5 [file sciadv.adk2998_sm.pdf]

Science Advances
AAAS

Supplementary Materials for

Biogeography of community canopy leaf traits and their links to global forest photosynthesis

Feng Jiang *et al.*

Corresponding author: Zhiheng Wang, zhiheng.wang@pku.edu.cn

Sci. Adv. **12**, eadk2998 (2026)
DOI: 10.1126/sciadv.adk2998

This PDF file includes:

Figs. S1 to S16
Tables S1 to S5

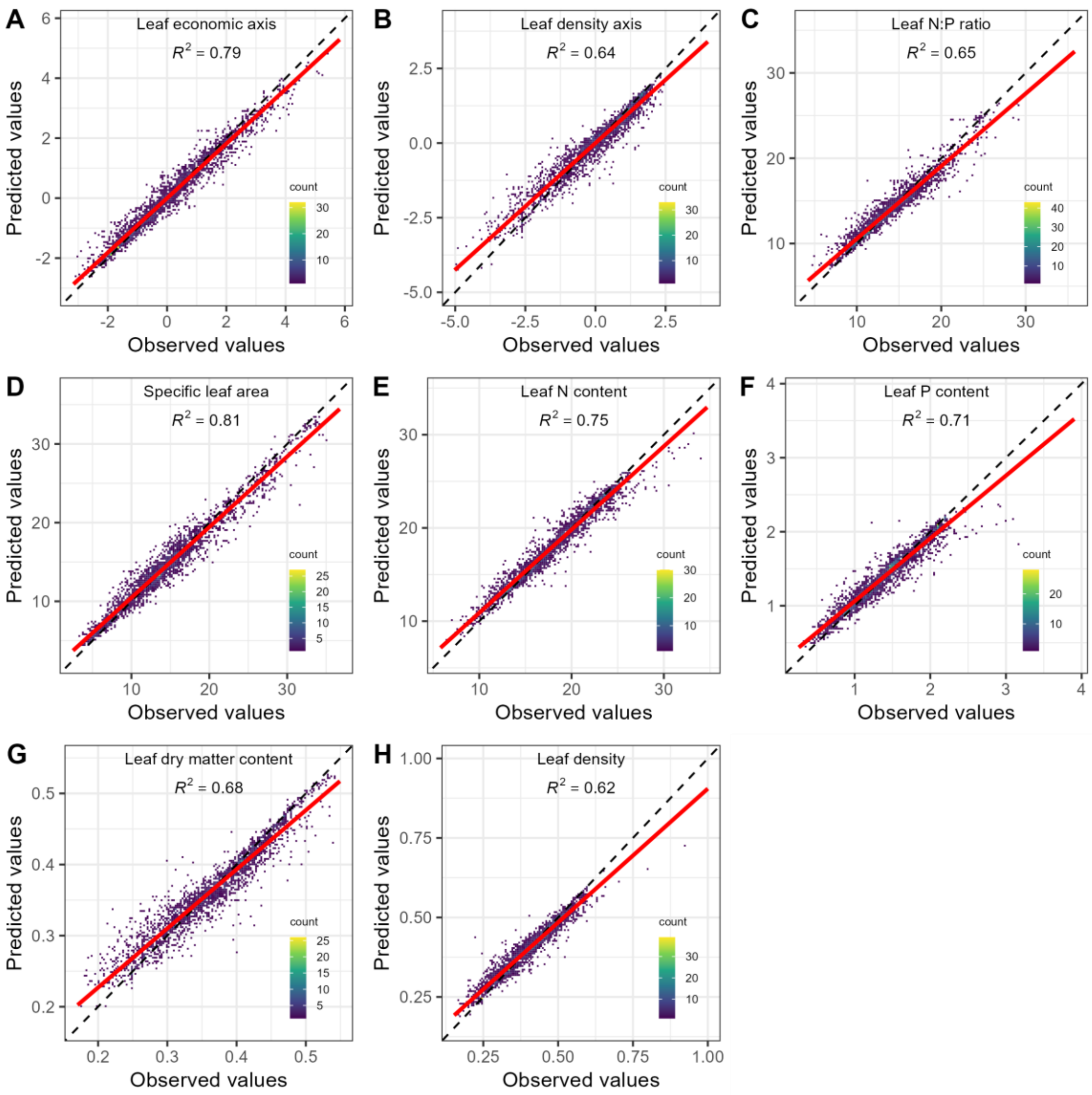

Fig. S1. Model performance of community canopy leaf traits.

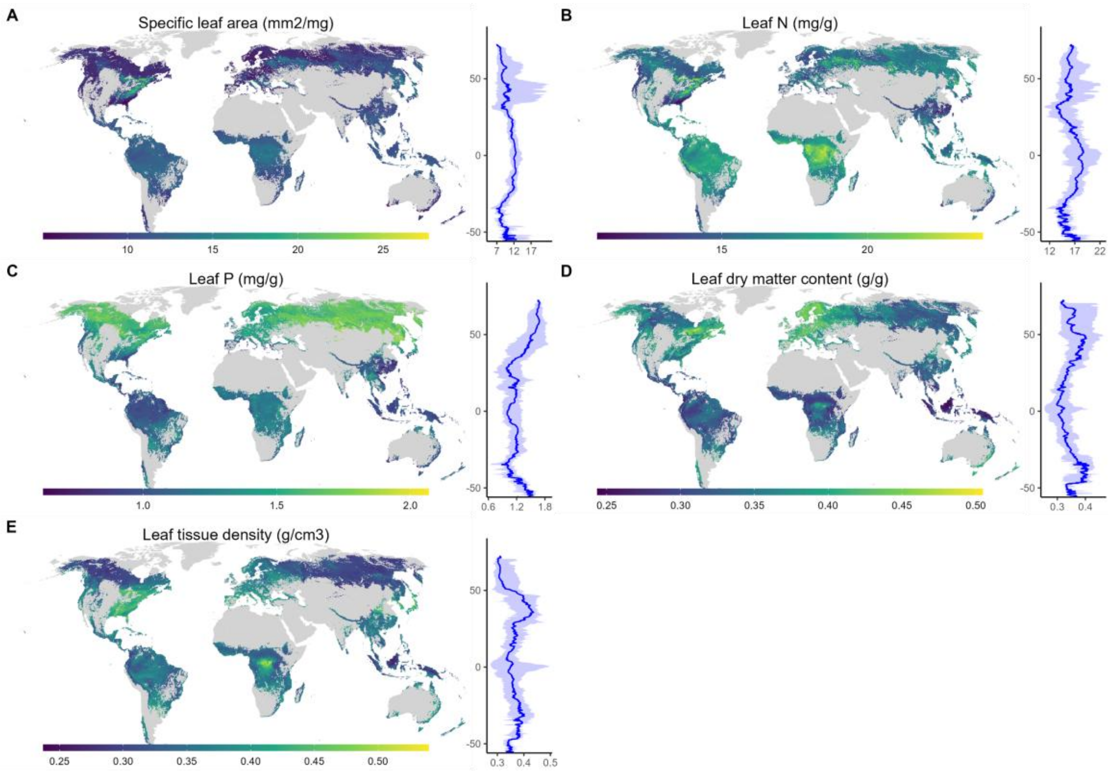

Fig. S2. Global distributions of community canopy leaf traits. Plots on the right of these panels show the latitudinal gradients of leaf traits (median with 5% and 95% quantiles).

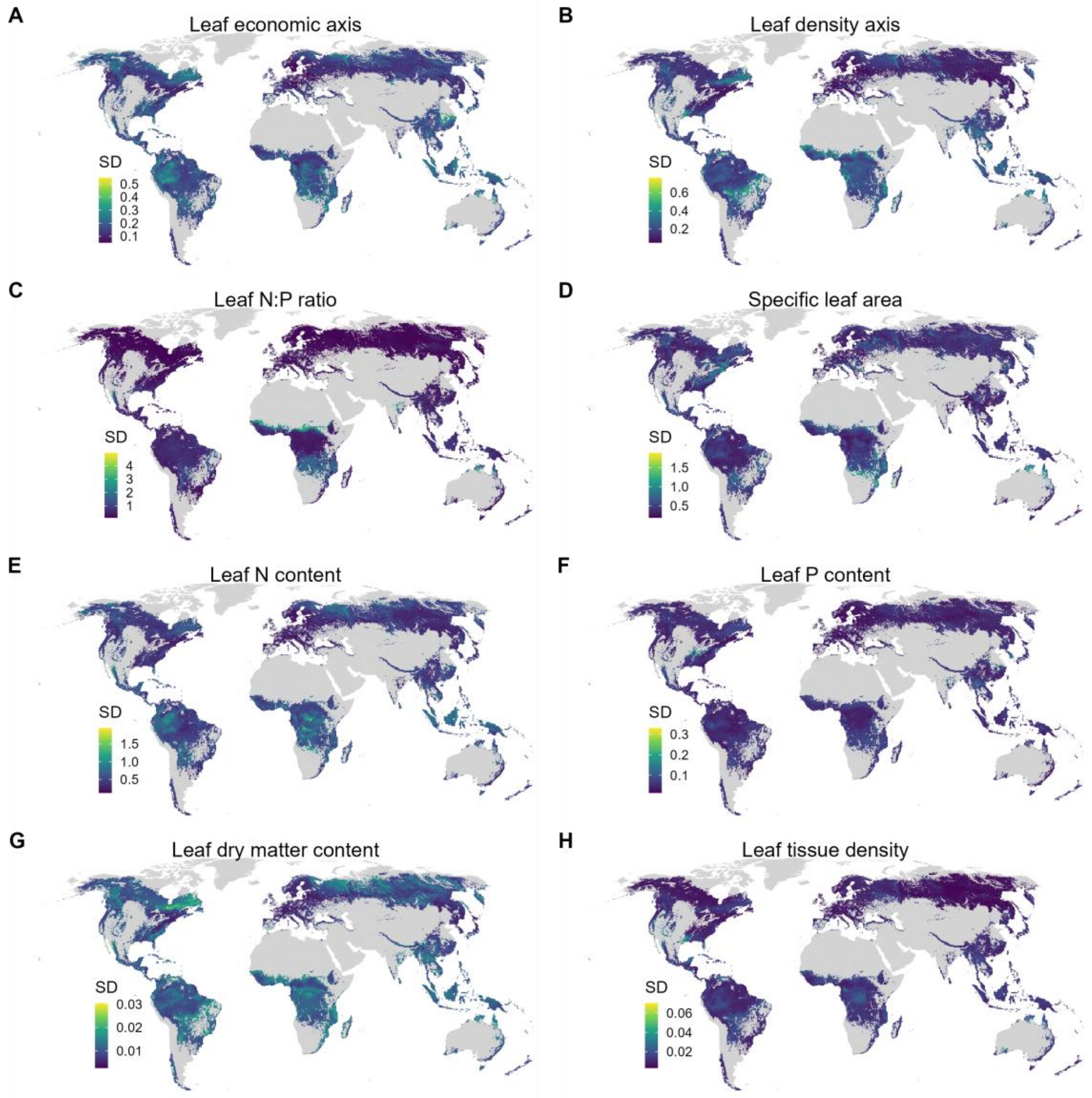

Fig. S3. Maps of the standard deviation for community canopy leaf traits.

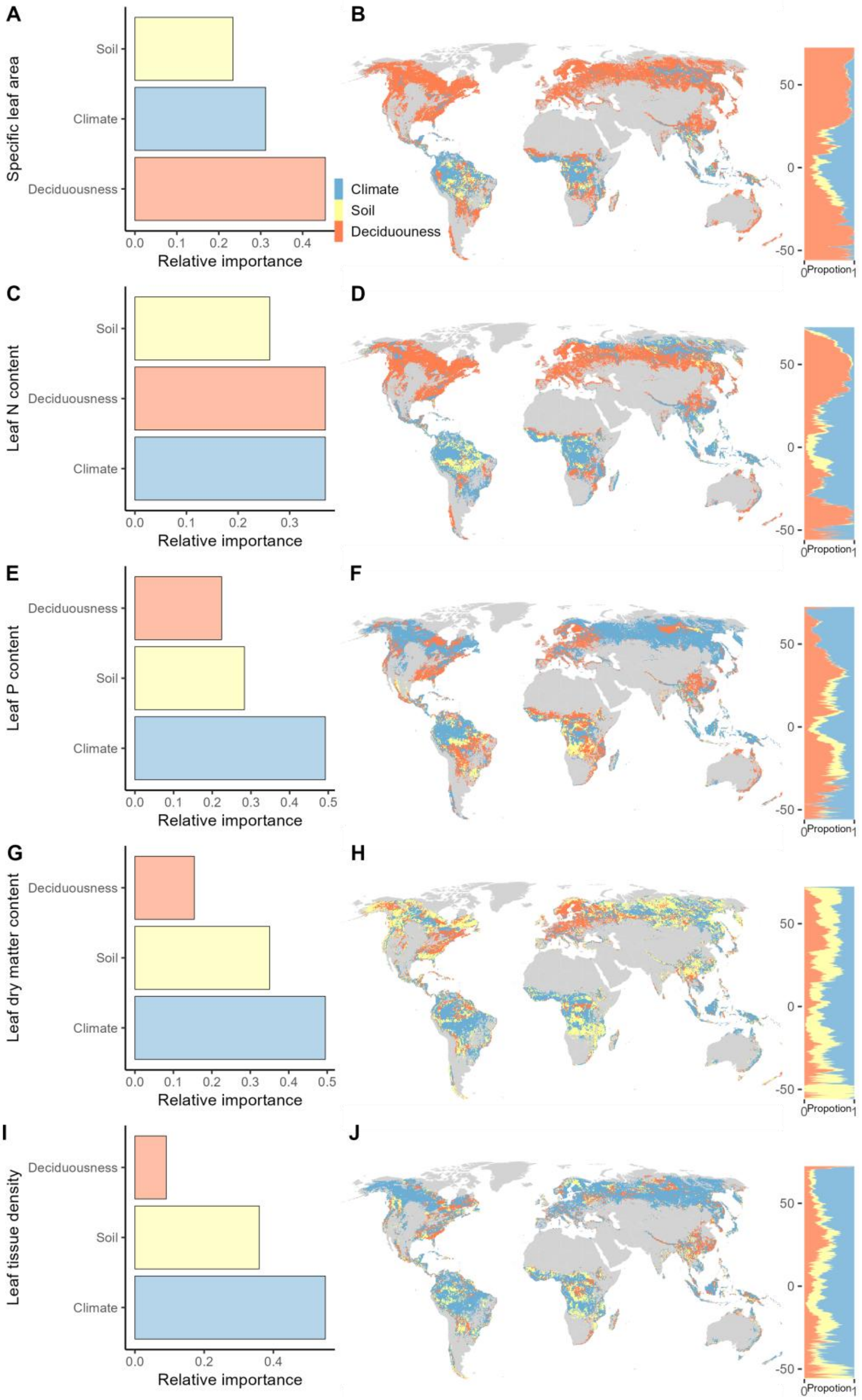

Fig. S4. Maps of the dominant drivers for community canopy leaf traits. **A, C, E, G, I,** relative importance of four predictor groups in random forest models (*N*=2,797). **B, D, F, H, J,** maps of the dominant drivers. Figures on the right of panels B, D, F, H, and J show the proportion of each dominant driver along with the latitudinal gradient (i.e., the number of grid cell for one dominant driver divided by the total number of grid cells in each 0.1-degree latitudinal bin).

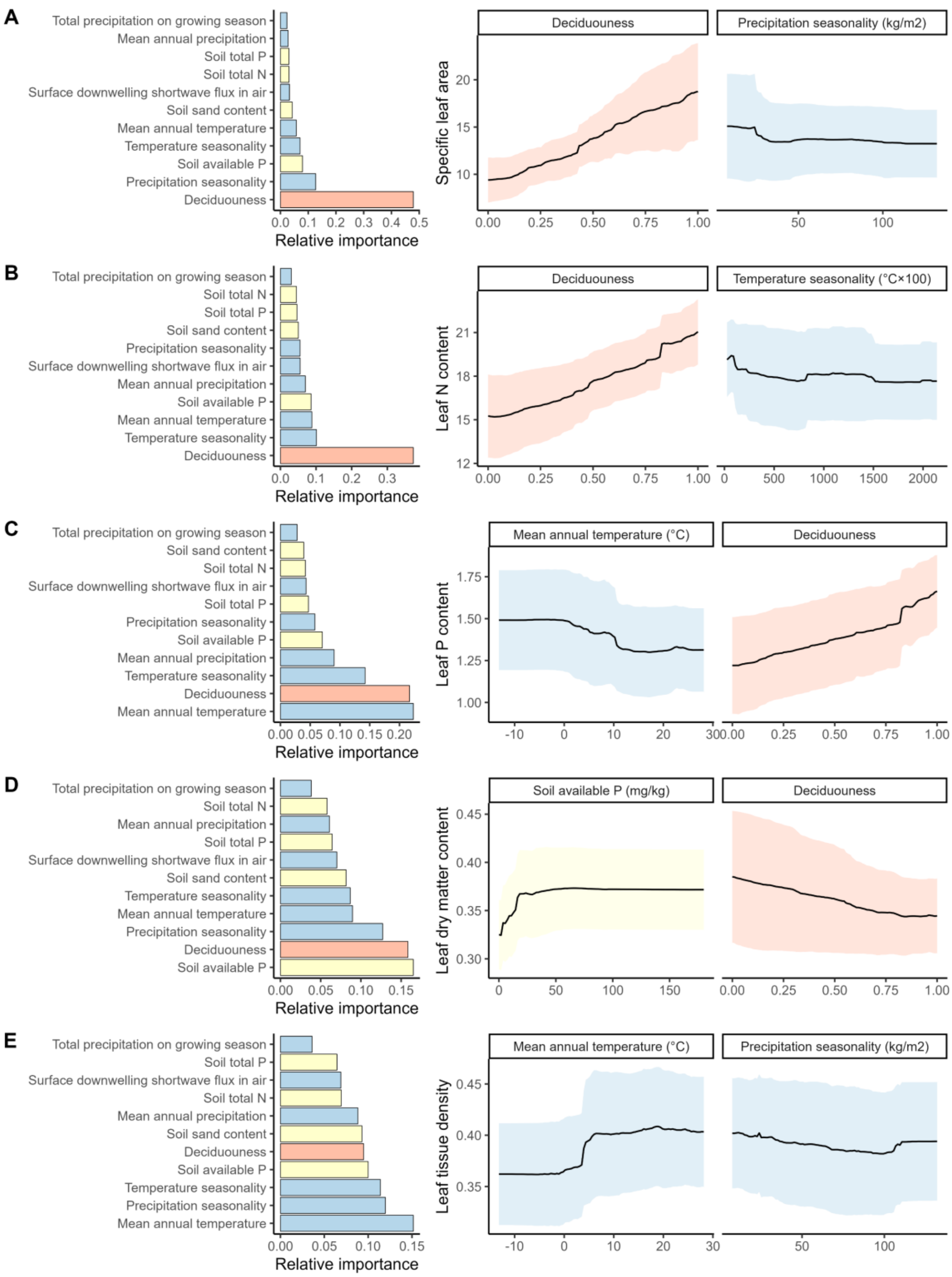

Fig. S5. Variable relative importance for community canopy leaf traits. Left panels show the relative importance of variables; right panels show the relationships between community canopy leaf traits and predictors (Partial dependence plot).

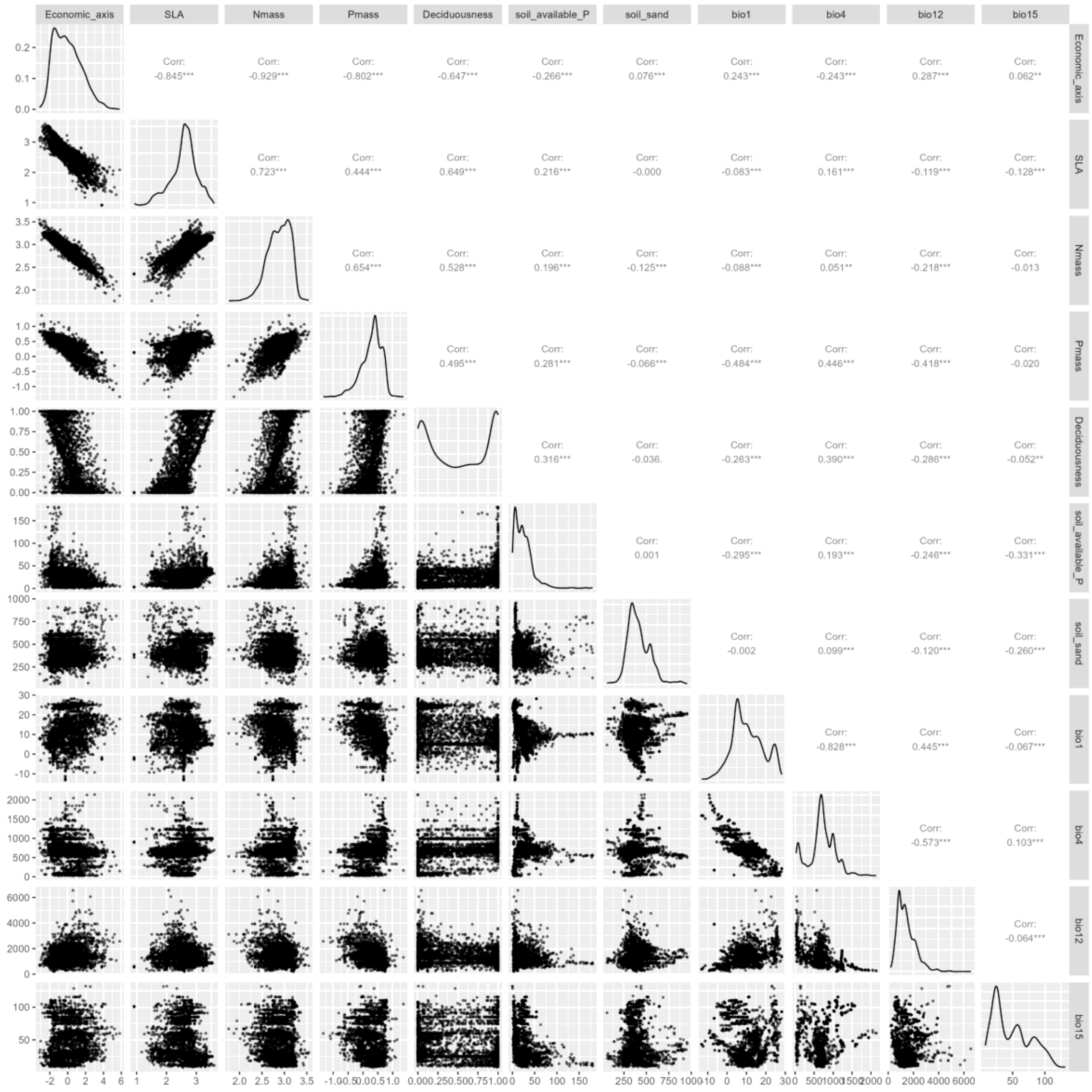

Fig. S6. Pairwise scatterplot matrix and corresponding correlations.

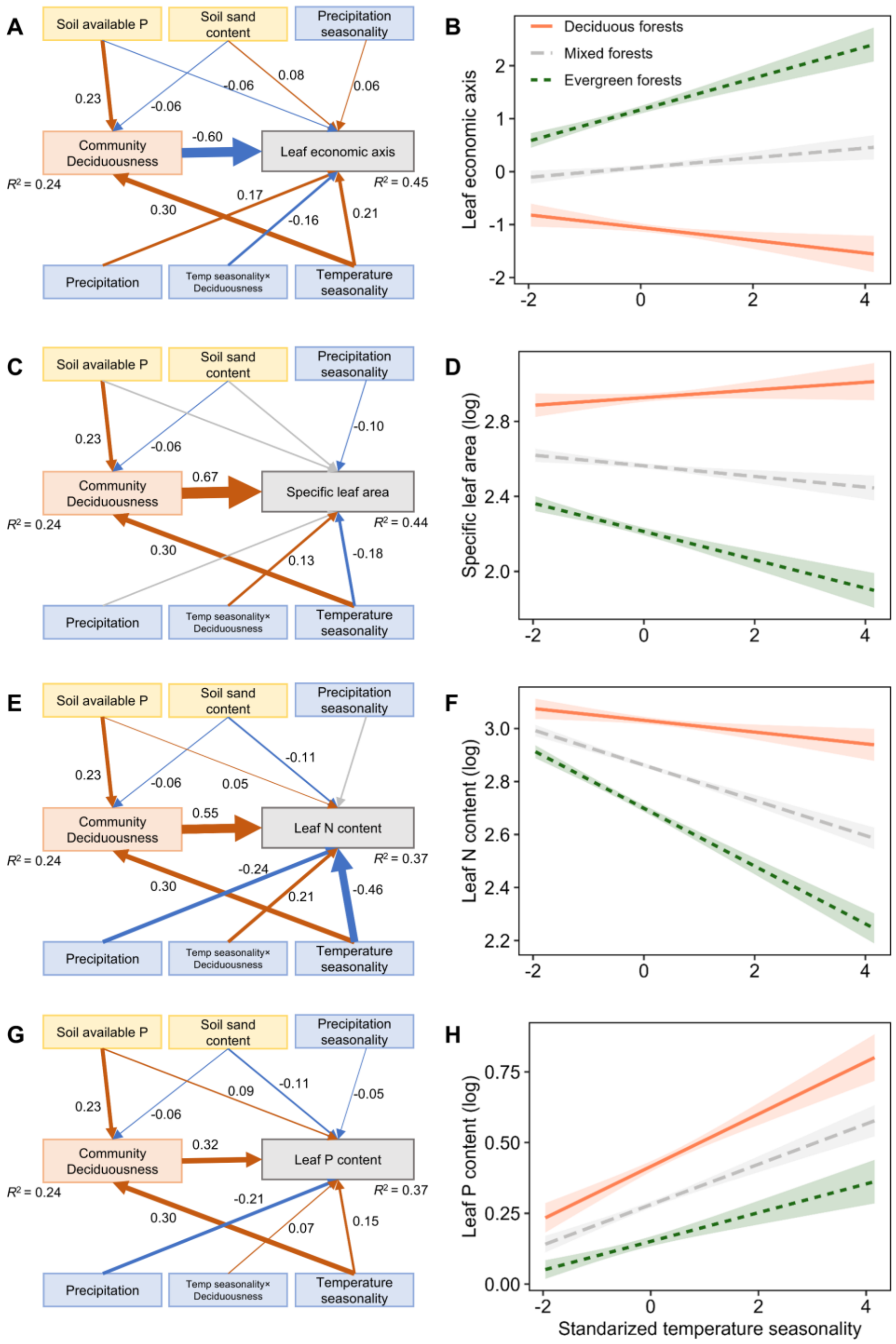

Fig. S7. Community deciduousness mediates environment–individual trait relationships. We use temperature seasonality in place of mean annual temperature. The models have a good fit (*Fisher's C* = 3.157, *P* = 0.206). **A, C, E, G,** structural equation modelling, leaf economic axis, characterized by lower specific leaf area and nitrogen content per unit dry mass; path coefficients are standardized: brown lines indicate significant positive relationships, blue lines significant negative relationships, and gray lines non-significant relationships (*P*>0.05). Line thickness reflects the strength of the effect. **B, D, F, H,** interaction plots show that the relationships between temperature seasonality and leaf traits vary across different levels of community deciduousness (0.04, 0.51, and 0.99).

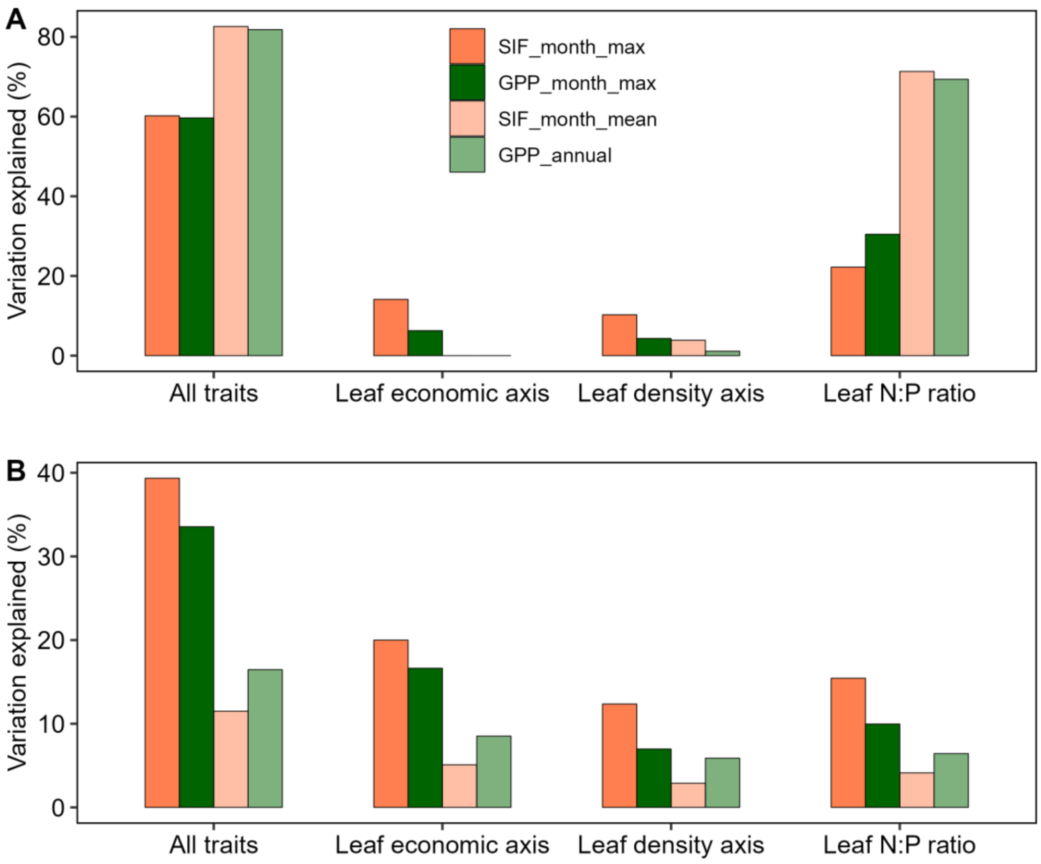

Fig. S8. Variation in SIF and GPP explained by leaf traits using random forest models. A, variation explained by traits without controlling for growing season length; R^2 values were obtained directly from the random forest models. **B,** variation explained by traits after controlling for growing season length; R^2 was calculated as the difference between models including both leaf traits and growing season length as predictors and models including only growing season length. "All traits" refers to models including all three trait dimensions simultaneously. SIF, monthly maximum of sun-induced fluorescence. GPP, gross primary productivity. To quantitatively support our choice of using peak photosynthetic capacity, we evaluated the predictive ability of plant traits for four SIF- and GPP-based metrics representing both annual means and maxima, including maximum monthly SIF, mean monthly SIF, annual GPP, and maximum monthly GPP.

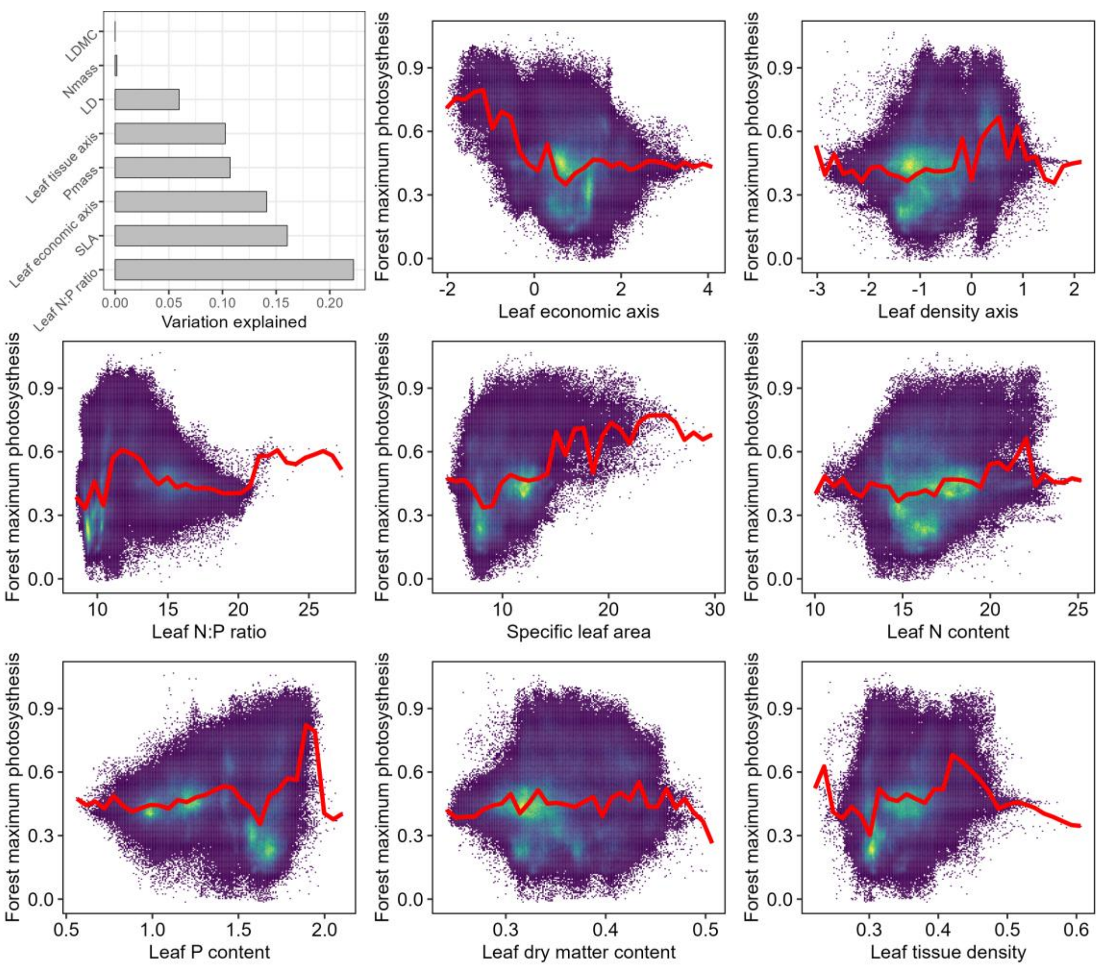

Fig. S9. Univariate relationship between forest maximum photosynthesis and each community canopy leaf trait. Forest maximum photosynthesis is represented by monthly maximum of sun-induced fluorescence (SIF). The variation in SIF values explained by each trait map was evaluated separately using random forest models. Red lines in the heatmaps represent the fitted relationships from the models.

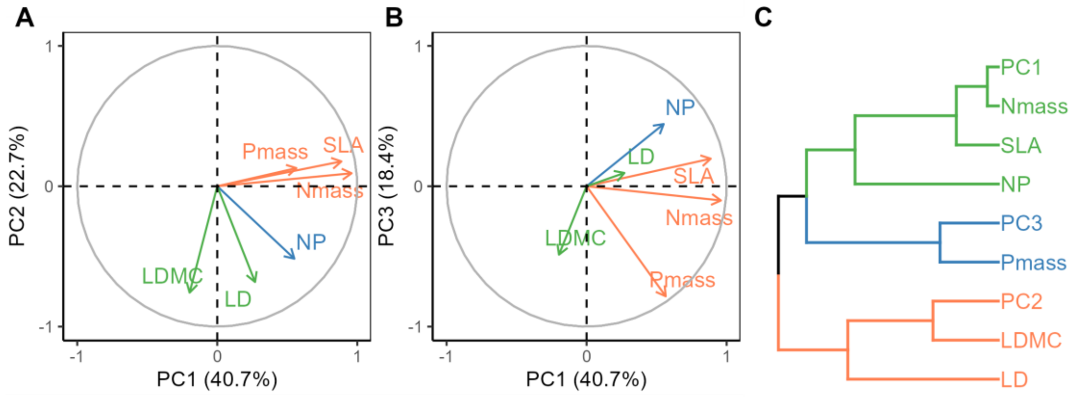

Fig. S10. PCA of community canopy traits based on non-imputing trait data. A, B, principal component analysis for community canopy leaf traits (*N*=1,026 plots which have all trait data); **C,** clustering of these traits together with the extracted principal components in panels A and B. All trait variables were log-transformed and standardized (z-scores) before principal component analysis.

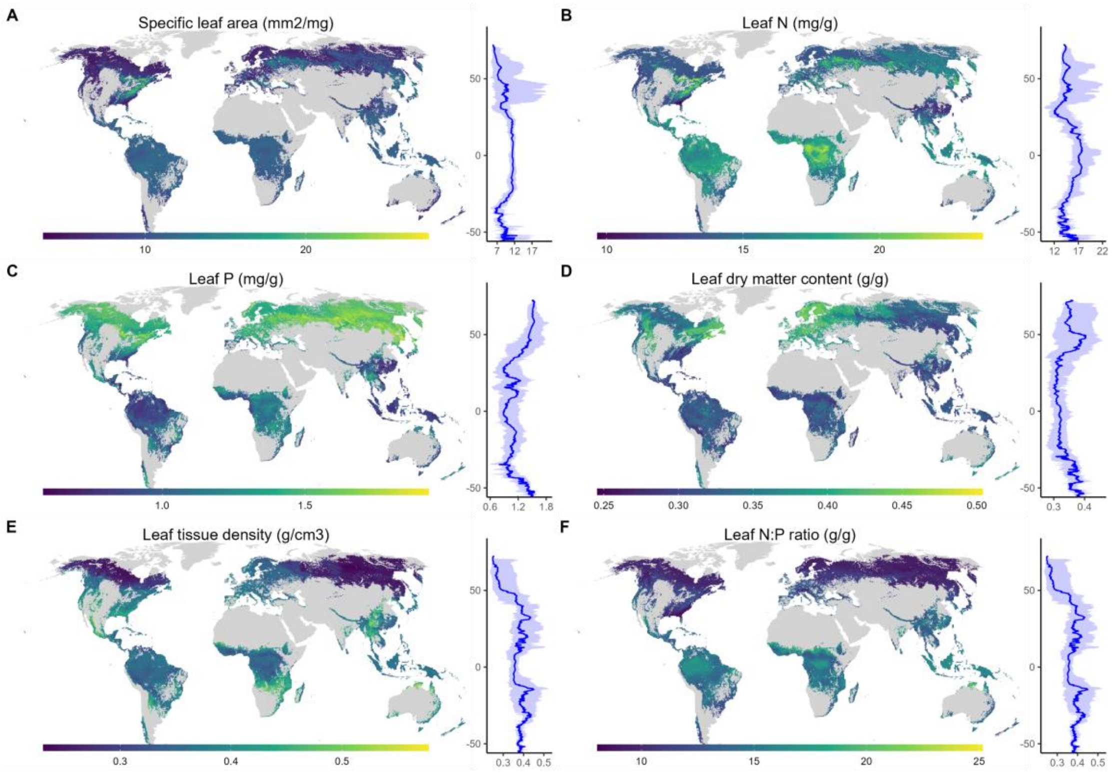

Fig. S11. Trait maps based on non-imputing trait data. Plots on the right of these panels show the latitudinal gradients of leaf traits (median with 5% and 95% quantiles).

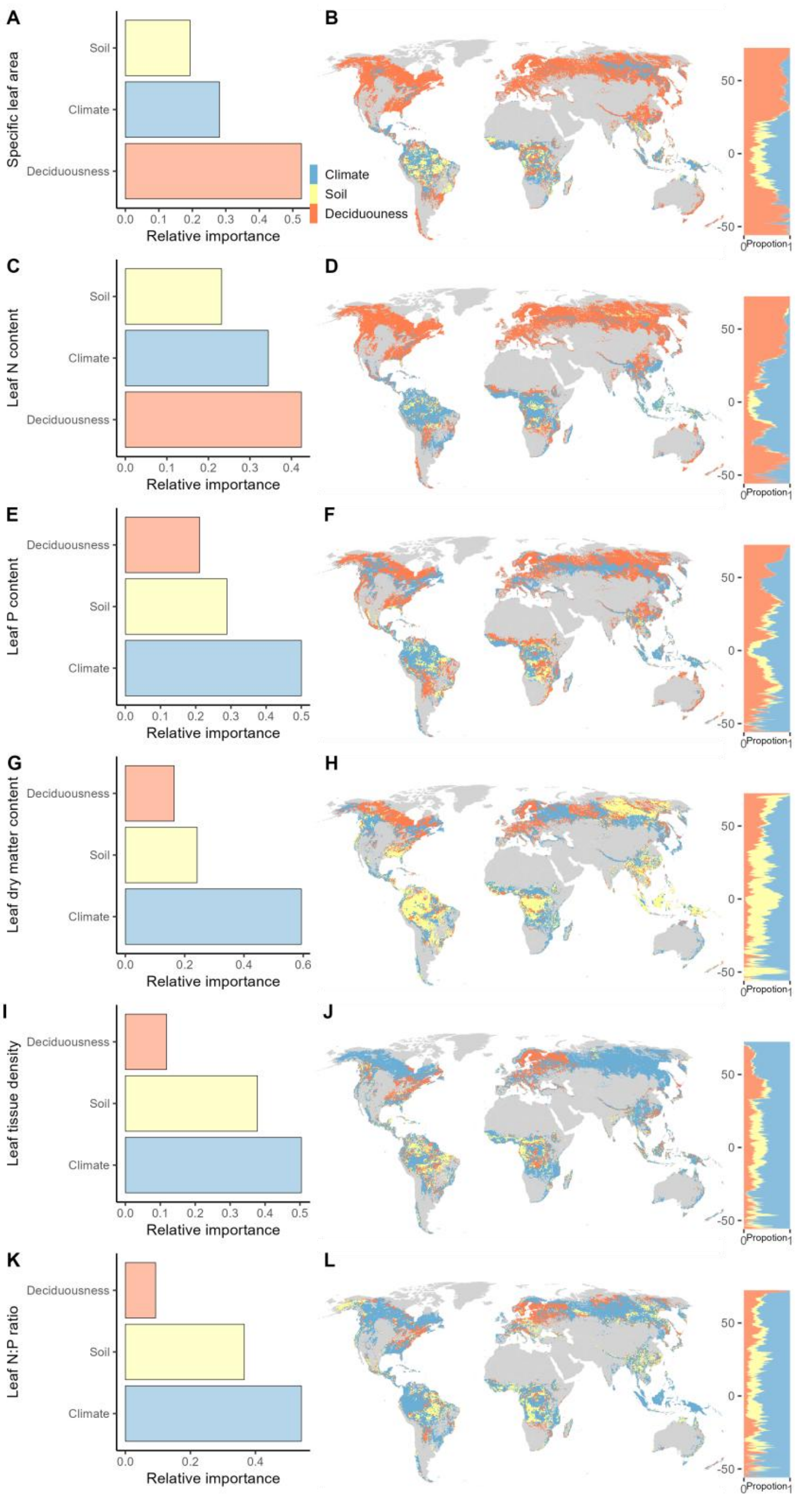

Fig. S12. Variable relative importance for predictor groups based on non-imputing trait data. A, C, E, G, I, K, relative importance of four predictor groups in random forest models. **B, D, F, H, J, L,** maps of the dominant drivers. Figures on the right of panels B, D, F, H, J and L show the proportion of each dominant driver along with the latitudinal gradient (i.e., the number of grid cell for one dominant driver divided by the total number of grid cells in each 0.1-degree latitudinal bin).

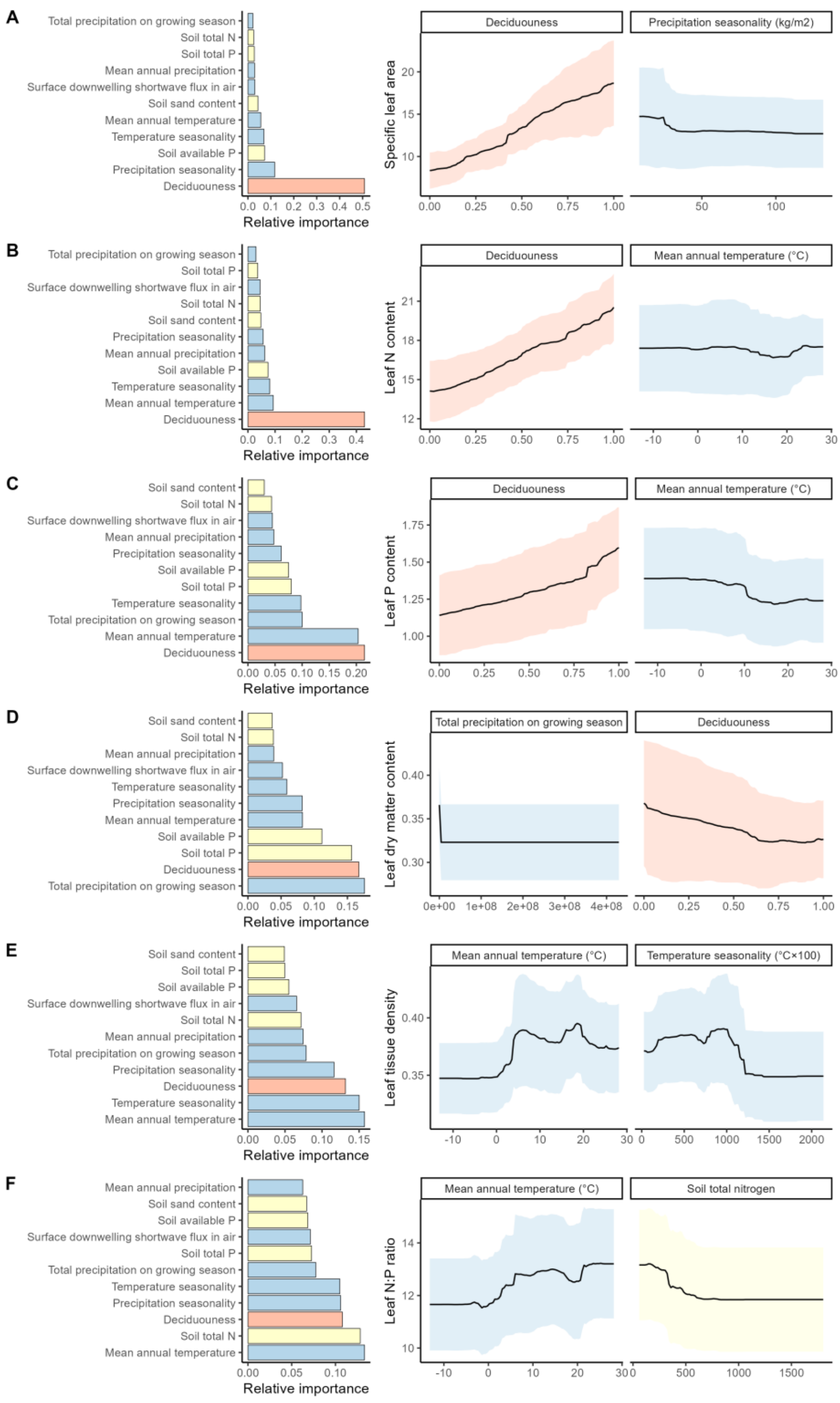

Fig. S13. Variable relative importance for individual variable based on non-imputing trait data. Left panels show the relative importance of variables; right panels show the relationships between community canopy leaf traits and predictors (Partial dependence plot).

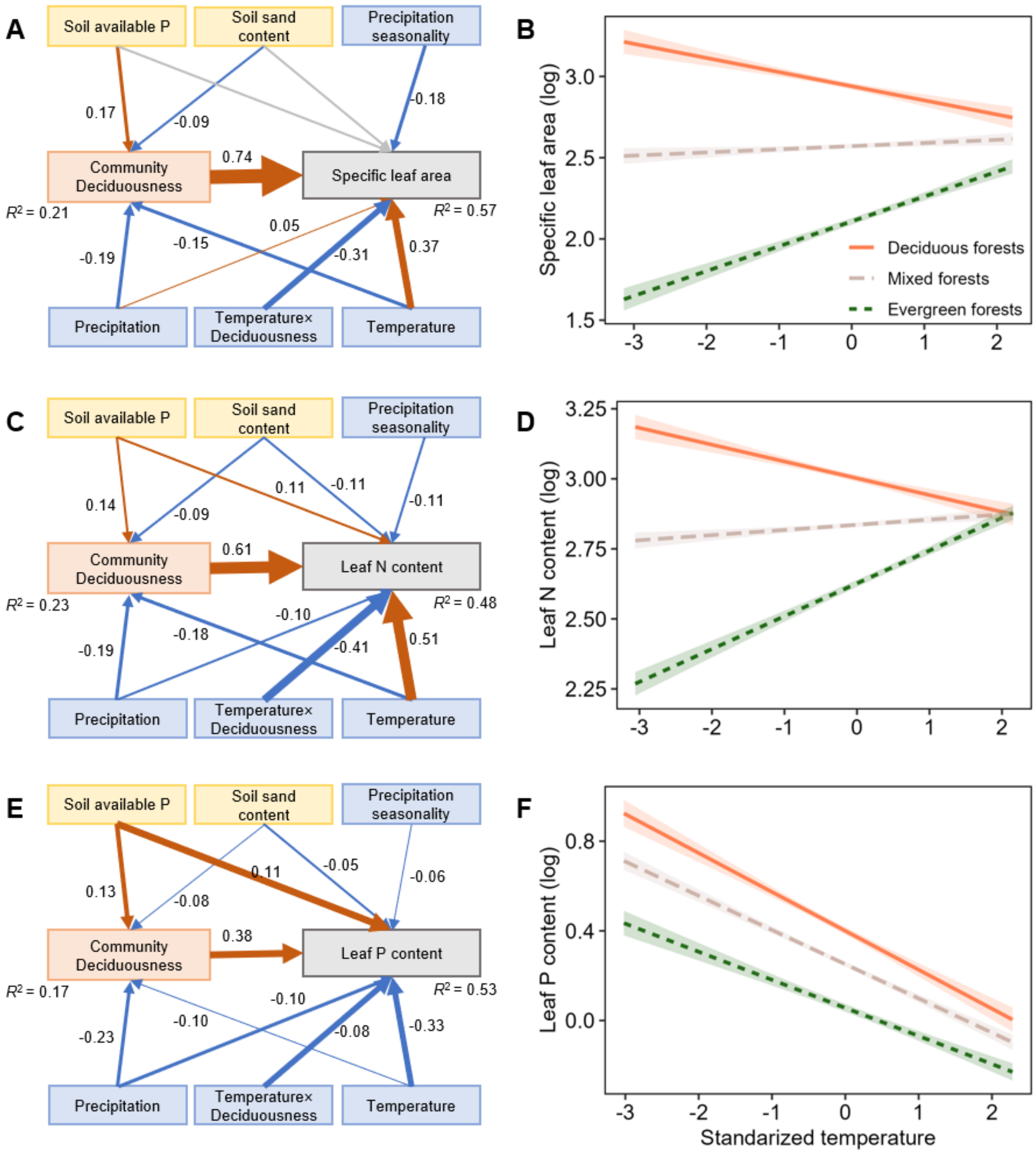

Fig. S14. Community deciduousness mediates environment–trait relationships based on non-imputing trait data. Our models have a good fit (A, *Fisher's C* = 3.915, *P* = 0.141; C, *Fisher's C* = 3.891, *P* = 0.143; E, *Fisher's C* = 0.769, *P* = 0.681). **A, C, E,** structural equation models; path coefficients are standardized: brown lines indicate significant positive relationships, blue lines significant negative relationships, and gray lines non-significant relationships (P>0.05). Line thickness reflects the strength of the effect. **B, D, F,** interaction plots show that the relationships between temperature and leaf traits vary across different levels of community deciduousness.

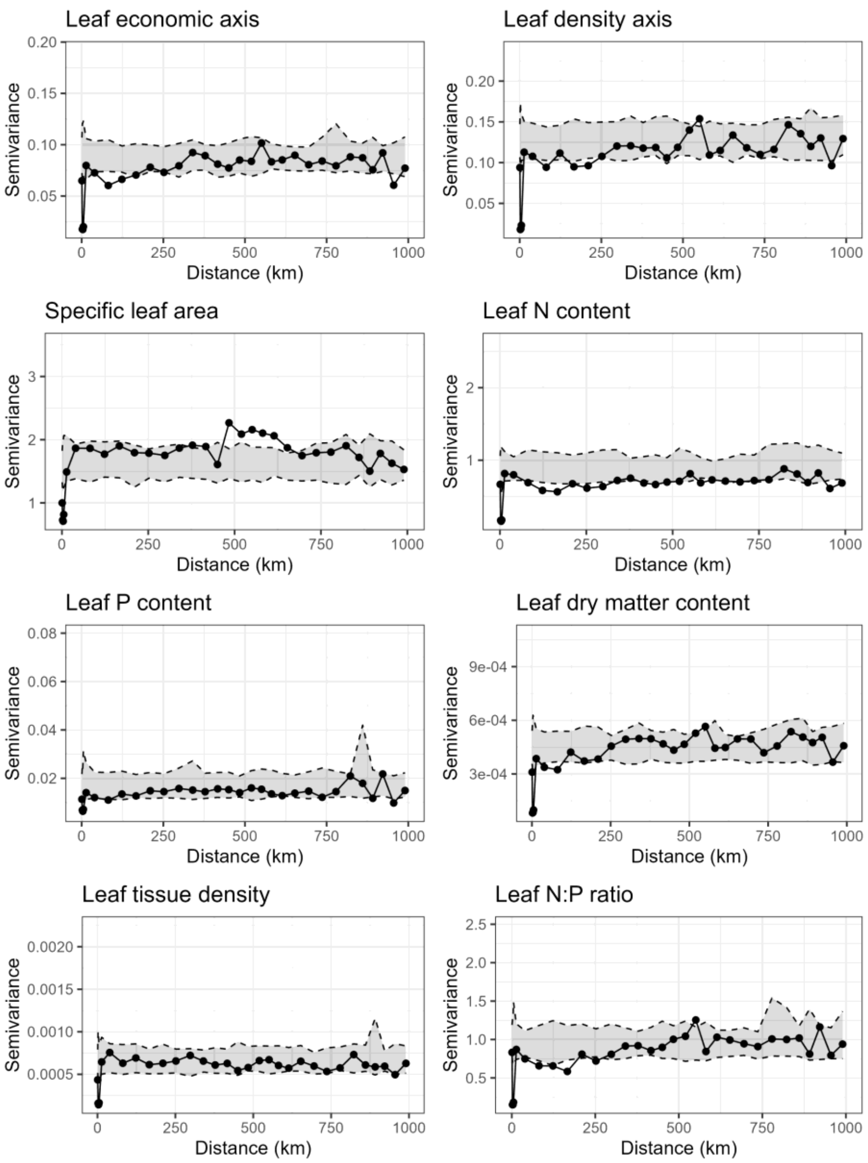

Fig. S15. Semivariograms showing the spatial autocorrelation of model residuals for different traits in the random forest models used for geo-mapping. The grey zones indicate the 95% confidence interval; points outside the envelope suggest semivariance values that differ significantly from expectations under spatial randomness, indicating strong spatial structure.

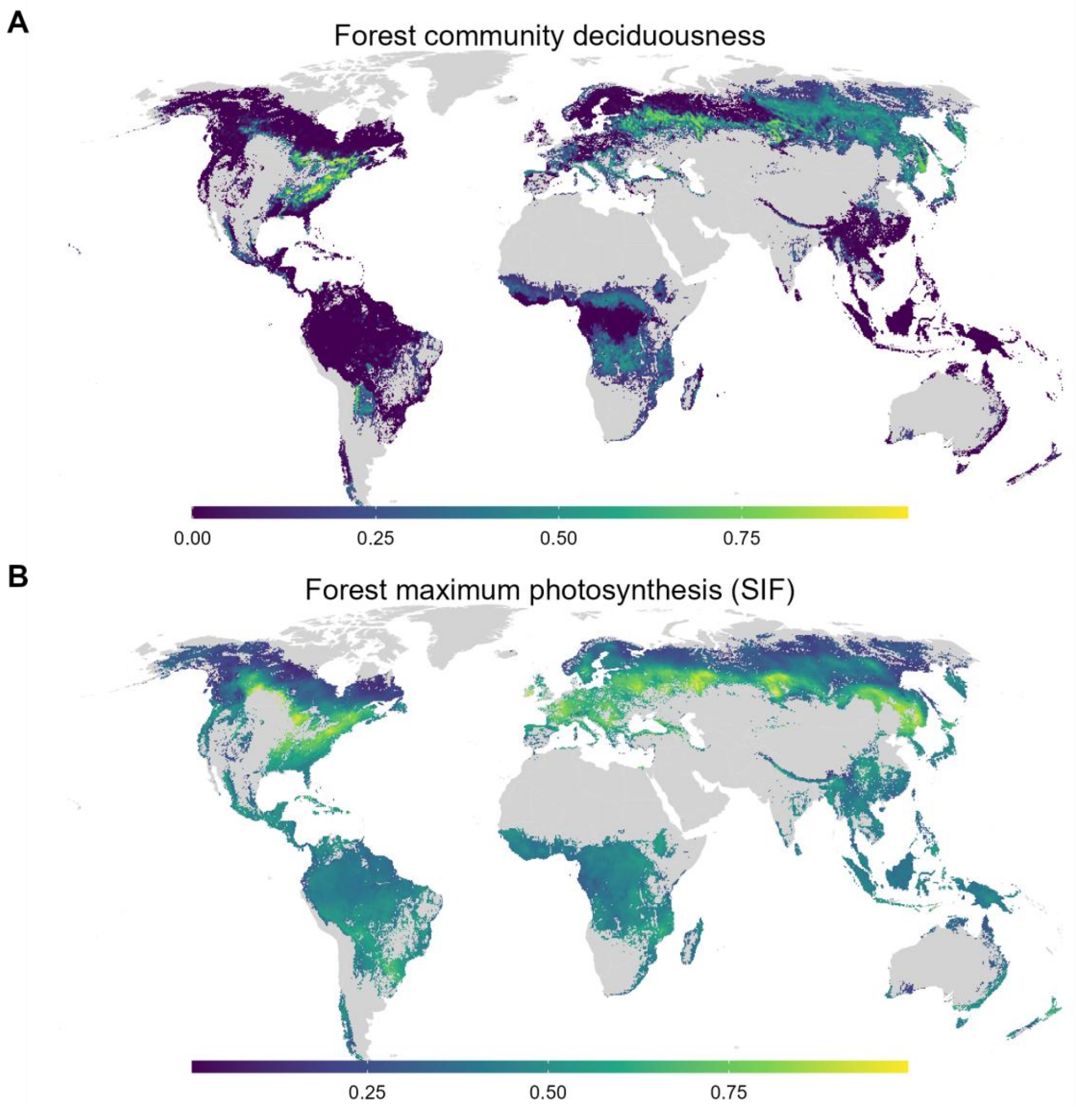

Fig. S16. Maps of forest community deciduousness and maximum photosynthesis. Forest community deciduousness was obtained from Harper et al. (2023) (*79*), and maximum photosynthesis was obtained from Zou et al. (2025) (*49*).

Table S1. **Pairwise correlations between community canopy leaf traits and the first three principal component (PC) axes of all leaf traits.**

	SLA	Nmass	Pmass	NP	LDMC	LD	PC1	PC2	PC3
SLA	1.00	0.72	0.44	0.18	-0.09	0.27	-0.86	0.10	0.23
Nmass		1.00	0.65	0.25	-0.08	0.14	-0.91	0.19	0.21
Pmass			1.00	-0.45	**-0.02**	0.06	-0.71	0.46	-0.47
NP				1.00	0.17	0.27	-0.16	-0.58	0.75
LDMC					1.00	0.70	-0.14	-0.81	-0.49
LD						1.00	-0.43	-0.77	-0.30
PC1							1.00	**-0.01**	**0.01**
PC2								1.00	**0.02**
PC3									1.00

Note: Bold values indicate non-significant correlations ($P > 0.05$), whereas all other correlations are significant ($P < 0.05$).

Table S2. Sample size for different biomes.

Biomes	Plot number
Boreal Forests/Taiga	45
Deserts & Xeric Shrublands	4
Mangroves	10
Mediterranean Forests, Woodlands & Scrub	139
Montane Grasslands & Shrublands	75
Temperate Broadleaf & Mixed Forests	1422
Temperate Conifer Forests	300
Temperate Grasslands, Savannas & Shrublands	227
Tropical & Subtropical Dry Broadleaf Forests	24
Tropical & Subtropical Grasslands, Savannas & Shrublands	25
Tropical & Subtropical Moist Broadleaf Forests	507
Tundra	8
Unknown	11

Table S3. Proportions of imputed plots and species.

Traits	Plots using imputed trait data [1]	Plots using only non-imputed trait data [2]	Proportion of imputed plots (%) [3]	Proportion of imputed species (%) [4]
SLA	2797	2467	11.8	30.7
Nmass	2797	2505	10.4	31.3
Pmass	2797	2400	14.2	50.3
LDMC	2797	1809	35.3	68.7
LD	2797	1301	53.5	70.9
N:P ratio	2797	1891	32.4	73.4

[1] All plots number used in our main text.

[2] If we do not impute species traits, some plots will be removed because large number of trees (> 40% individuals) have no trait values matched.

[3] Proportion of plots have been removed if we do not impute species traits.

[4] Proportion of species have been imputed before we calculated community canopy trait values.

Table S4. Predictors used in this study.

Group	Source	Resolution	Variables
Climate	Chelsa	30s	Mean annual temperature
Climate	Chelsa	30s	Mean diurnal temperature range
Climate	Chelsa	30s	Isothermality
Climate	Chelsa	30s	Temperature seasonality
Climate	Chelsa	30s	Mean daily maximum air temperature of the warmest month
Climate	Chelsa	30s	Mean daily minimum air temperature of the coldest month
Climate	Chelsa	30s	Annual range of air temperature
Climate	Chelsa	30s	Mean daily mean air temperatures of the wettest quarter
Climate	Chelsa	30s	Mean daily mean air temperatures of the driest quarter
Climate	Chelsa	30s	Mean daily mean air temperatures of the warmest quarter
Climate	Chelsa	30s	Mean daily mean air temperatures of the coldest quarter
Climate	Chelsa	30s	Mean annual precipitation
Climate	Chelsa	30s	Precipitation amount of the wettest month
Climate	Chelsa	30s	Precipitation amount of the driest month
Climate	Chelsa	30s	Precipitation seasonality
Climate	Chelsa	30s	Mean monthly precipitation amount of the wettest quarter
Climate	Chelsa	30s	Mean monthly precipitation amount of the driest quarter
Climate	Chelsa	30s	Mean monthly precipitation amount of the warmest quarter
Climate	Chelsa	30s	Mean monthly precipitation amount of the coldest quarter
Climate	Chelsa	30s	Growing degree days heat sum above 5°C
Climate	Chelsa	30s	Growing season length
Climate	Chelsa	30s	Mean temperature of the growing season
Climate	Chelsa	30s	Total precipitation amount on growing season days
Climate	Chelsa	30s	Vapor pressure deficit
Climate	Chelsa	30s	Surface downwelling shortwave radiation
Climate	Zomer et al. 2022 SD	30s	Aridity index
Climate	Zomer et al. 2022 SD	30s	Potential evapotranspiration
Soil	Soilgrids250m	30s	Bulk density
Soil	Soilgrids250m	30s	Soil cation exchange capacity
Soil	Soilgrids250m	30s	Coarse fragments
Soil	Soilgrids250m	30s	Soil total nitrogen
Soil	Soilgrids250m	30s	Soil pH
Soil	Soilgrids250m	30s	Soil organic carbon
Soil	Soilgrids250m	30s	Soil organic carbon density
Soil	Soilgrids250m	30s	Soil sand content
Soil	Soilgrids250m	30s	Soil silt content
Soil	Soilgrids250m	30s	Soil clay content
Soil	Soilgrids250m	30s	Soil volumetric water
Soil	McDowell et al. 2023	30s	Soil available phosphorus
Soil	He et al. 2021 ESSD	0.5 degree	Soil total phosphorus
Topography	Amatulli et al. 2018 SD	30s	Elevation
Topography	Amatulli et al. 2018 SD	30s	Slope
Community deciduousness for mapping	Harper et al. 2023	300m	Proportion of deciduous trees

Table S5. Model performance under spatial cross-validation with varying buffer distances

Trait	Fold	Train R^2	Cross-validation R^2
Leaf economic axis	Clust_10km	0.79	0.68
	Clust_50km	0.79	0.63
	Clust_100km	0.79	0.62
Leaf density axis	Clust_10km	0.65	0.51
	Clust_50km	0.65	0.41
	Clust_100km	0.65	0.39
SLA	Clust_10km	0.81	0.76
	Clust_50km	0.81	0.59
	Clust_100km	0.81	0.58
Nmass	Clust_10km	0.76	0.63
	Clust_50km	0.76	0.62
	Clust_100km	0.76	0.60
Pmass	Clust_10km	0.71	0.57
	Clust_50km	0.71	0.52
	Clust_100km	0.71	0.51
LDMC	Clust_10km	0.69	0.56
	Clust_50km	0.69	0.46
	Clust_100km	0.69	0.44
LD	Clust_10km	0.63	0.49
	Clust_50km	0.63	0.33
	Clust_100km	0.63	0.31
N:P ratio	Clust_10km	0.66	0.44
	Clust_50km	0.66	0.40
	Clust_100km	0.66	0.38
